# Optimal parameter determination of repetitive transcranial magnetic stimulation for treating treatment-resistant depression: A network meta-analysis of randomized controlled trials

**DOI:** 10.3389/fpsyt.2022.1038312

**Published:** 2022-12-01

**Authors:** Jinbiao Li, Liqian Cui, Hao Li

**Affiliations:** ^1^Department of Neurology, The First Affiliated Hospital, Sun Yat-sen University, Guangzhou, China; ^2^Guangdong Provincial Key Laboratory of Diagnosis and Treatment of Major Neurological Diseases, National Key Clinical Department and Key Discipline of Neurology, Guangzhou, China

**Keywords:** repetitive transcranial magnetic stimulation, treatment-resistant depression, network meta-analysis, randomized controlled trials, optimal parameter

## Abstract

**Background:**

Many studies have shown the efficacy of repetitive transcranial magnetic stimulation (rTMS) in treatment-resistant depression (TRD). However, the choice of different parameters has been a challenging issue.

**Methods:**

PubMed, Cochrane, and Embase databases were searched for relevant studies until June 20, 2022. The treatment efficacy was evaluated by the relative risk (RR) using the pairwise test for response and remission rates. Subgroup and sensitivity analyses were conducted to explore the primary outcome differences and to assess the reliability of the results.

**Results:**

Thirty-seven trials comprising 2120 participants with TRD were included. The more efficacious interventions compared to sham controls included high-frequency left followed by low-frequency right sup-threshold (HFL-LFR-sup-rTMS, RR = 5.29, 95% CI: 1.24–22.50), high-frequency left sup-threshold (HFL-sup-rTMS, RR = 2.97, 95% CI: 1.74–5.05), low-frequency right sup-threshold (LFR-sup-rTMS, RR = 2.72, 95% CI: 1.50–4.90), low-frequency right followed by high-frequency left sup-threshold (LFR-HFL-sup-rTMS, RR = 2.71, 95% CI: 1.62–4.53), and high-frequency left sub-threshold (HFL-sub-rTMS, RR = 1.91, 95% CI: 1.18–3.10) rTMS. The estimated relative ranking of treatments suggested that HFL-LFR-sup-rTMS (84.4%) might be the most efficacious among all rTMS strategies. No treatments showed a lower acceptability than the sham control.

**Limitations:**

Subgroup analysis was not conducted to compare the efficacy of rTMS treatment between bipolar and unipolar depression, and small-study effects possibly introduced bias.

**Conclusion:**

Treatment with HFL-LFR-sup-rTMS, HFL-sup-rTMS, LFR-sup-rTMS, LFR-HFL-sup-rTMS, or HFL-sub-rTMS is more efficacious than the sham control. HFL-LFR-sup-rTMS and HFL-sup-rTMS may be the two best among the most efficacious rTMS treatments.

**Systematic review registration:**

[https://www.crd.york.ac.uk/PROSPERO], identifier [CRD42022334481].

## Introduction

Major depressive disorder (MDD) is one of the common psychiatric disorders. The World Health Organization considers this disorder the third most common cause of worldwide disease, and by 2030, it is forecasted to be the most common (1–3). However, most patients with MDD fail to benefit from conventional antidepressant treatments. In some cases, inconsistencies in treatment success have been observed, including one or more, two or more, and three or more treatment failures (4). It has been reported that 67% of patients could achieve overall cumulative remission after two treatment steps (5).

In comparison, the chances of subsequent remission were much lower (36.8%, 30.6%, 13.7%, and 13.0% for the first, second, third, and fourth acute treatment steps, respectively), suggesting that the second step was a significant turning point (5). Thus, treatment-resistant depression (TRD) is most commonly defined as depression in patients having a minimum of two prior treatments with an inadequate response to a sufficient dose and duration of antidepressant (4). Therefore, the treatment of TRD has been a significant concern, and it is still a major therapeutic challenge to develop novel and effective treatments.

In this context, researchers have found physical therapy to be an efficacious alternative, with repetitive transcranial magnetic stimulation (rTMS) showing great potential. In recent years, studies have demonstrated the efficacy and acceptability of rTMS for treating TRD (6–11). rTMS is a non-invasive neuromodulatory therapy for MDD that is safe and tolerable, with a low incidence of adverse events (12). A systematic review and meta-analysis of 32 randomized controlled trials (RCTs), including unilateral or bilateral rTMS, have revealed that compared to sham groups, the pooled response rate for unilateral rTMS was 2.00 [95% confidence interval (CI): 1.26–3.19], favoring unilateral rTMS; and for bilateral rTMS and sham groups, the pooled response rate was 3.55 (95% CI: 1.87–6.76), favoring bilateral rTMS (13). In another study, 47 RCTs were selected to perform the network meta-analysis (NMA), and the findings demonstrated that low-frequency right rTMS (LFR-rTMS, RR = 2.97, 95% CI: 1.61–5.49), high-frequency left rTMS (HFL-rTMS, RR = 2.91, 95% CI: 1.48–3.23), and bilateral rTMS (BL-rTMS, RR = 3.29, 95% CI: 1.88–5.73) have a higher efficacy than the sham control (14). rTMS also had a non-inferior acceptability compared to the sham control, and bilateral rTMS was more acceptable than bitemporal electroconvulsive therapy (BT-ECT, RR = 0.18, 95% CI: 0.03–0.89) (14).

Many meta-analyses have supported the efficacy and acceptability of rTMS (LFR-rTMS, HFL-rTMS, and bilateral rTMS) vs. the sham control, and the selection of rTMS parameters, including the frequency, intensity, intertrain interval, and number of pulses per session, has a key role in its treatment (14–17). However, there is no definitive consensus regarding the optimal parameters for rTMS treatment. Moreover, previous meta-analyses usually did not distinguish in detail between the different parameters of rTMS (14, 16). In addition, several small RCTs have shown different efficacy of rTMS with other parameters. Thus, it is essential to determine the optimal parameters of rTMS and the intervention duration for treating TRD. The selection of the optimal parameters of rTMS is principally based on the stimulation sites, frequency, intensity, and treatment duration and has gained the attention of many researchers (13, 14, 16). Furthermore, NMA makes it possible to access the comparative efficacy, summarize the evidence, and analyze the merits of the various interventions. Therefore, this study aimed to perform a systematic review and NMA to determine the optimal parameters of rTMS for treating TRD.

## Methods

### Search strategy and selection criteria

A systematic review and NMA were performed according to the guidelines of the Preferred Reporting Items for Systematic Reviews and Meta-Analyses (PRISMA) statement and its extension (18, 19). Various published literature reports that included RCTs were searched using the PubMed, Cochrane Central Register of Controlled Trials, and EMBASE databases from their inception to June 20, 2022. Different frequencies, locations of the coil, intensities (the percentage of the resting motor threshold; RMT), and treatment durations of rTMS to treat adults with TRD were considered while searching the literature. When screening the trials, careful measures were taken to search only selected published literature by applying the key medical subject headings or a combination of their text words like “transcranial magnetic stimulation” and “depressive disorder, treatment-resistant” (Supplementary Table 1). Full-text articles, both peer-reviewed and non-peer-reviewed in English, were retrieved and evaluated by two independent reviewers (JL and HL) who performed the search; any disagreements were resolved through discussion with a third reviewer (LC).

**FIGURE 1:**
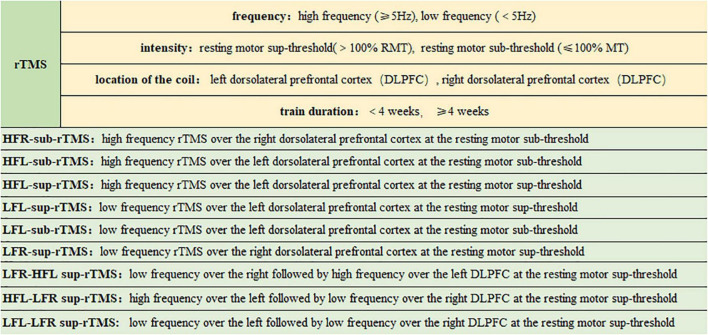
The classification of rTMS interventions analyzed in this study.

Randomized controlled trials (RCTs) of different parameter selections of rTMS used to treat TRD were included, and the following study characteristics were recorded: (1) Participant: Participants were limited to adults (over 18 years; both females and males) with a primary diagnosis of TRD including unipolar or bipolar depressive episodes and without secondary mood disorders, according to standard diagnostic criteria (DSM-IV, DSM-IV-TR, and ICD-10) (20). TRD was defined for MDD as failing to respond to at least two adequate doses and durations of antidepressant medications (4). (2) Intervention: Trials that have conducted at least two of the following interventions were included: Usually, in clinical practice, two significant modalities of rTMS are applied, i.e., low frequency and high frequency. However, both have specific functional activities; for example, rTMS with a high frequency (HF) and a low frequency (LF) are thought to impart a stimulating and inhibitory effect on the cerebral cortex, respectively. RMT > 100% was defined as sup-threshold rTMS (sup-rTMS), while RMT ≤ 100% defined as sub-threshold rTMS (sub-rTMS). Low frequency is graded as <5 Hz rTMS over the right dorsolateral prefrontal cortex (DLPFC) target at a delivered RMT > 100% (LFR-sup-rTMS). High frequency is graded as ≥5 Hz rTMS over the left DLPFC at a delivered RMT ≤ 100% (HFL-sub-rTMS) (21, 22). HFR-sub-rTMS, HFL-sup-rTMS, LFL-sup-rTMS, LFL-sub-rTMS, and LF stimulation were delivered to the right, followed by HF stimulation over the left DLPFC at the resting motor sup-threshold (LFR-HFL-sup-rTMS), HF over the left followed by LF over the right DLPFC at the resting motor sup-threshold (HFL-LFR-sup-rTMS), LF over the left followed by LF over the right DLPFC at the resting motor sup-threshold (LFL-LFR-sup-rTMS), and the sham control (Figure 1). (3) Comparison: The control group was restricted to another active treatment or the sham control. (4) Outcome: From all included studies, the main focus was to check the response rate of the treatment. Also, evaluation of depressive symptoms in TRD patients was performed with either the Hamilton Depression Rating Scale, 17 items, 24 items, or 28 items (HAMD-17, HAMD-24, and HAMD-28) or the Montgomery Asberg Depression Rating Scale (MARDS). (5) Study design: RCTs and randomized crossover trials were included, but only the first-phase data from crossover trials were obtained (23). Simultaneously, non-randomized controlled trials, quasi-randomized trials, and incomplete trials with only up to 20% of data or enrolled participants with secondary mood disorders like post-stroke depression or psychotic depression with Parkinson’s disease and other concomitant severe medical illnesses were excluded (20). (6) Papers published in a language other than English were excluded (14). The first and third authors (JL and HL) independently selected the studies, and all authors (JL, HL, and LC) discussed and resolved disagreements.

### Data extraction and outcome measures

Data were initially gathered by the first and third authors (JL and HL) independently; later, all authors (JL, HL, and LC) scrutinized, discussed, and resolved any disagreements and finalized the studies. The following predefined hierarchical characteristics, which are the essential factors according to the study, are presented in Supplementary Table 2: (1) Sources: Careful screening was done on the gathered data to report the details like the first author, publication year, and so on; (2) Details: Details regarding the ethnicity of the population were studied along with a clear period or duration of the study. Furthermore, demographic and clinical details like the sample size, mean age, and medication details, whether the patients are taking antidepressant drugs or not, intervention measures like rTMS frequency, location of the coil, intension, number of pulses per session, intervention duration, and depression scale were documented carefully; (3) Results: In-depth observation and recording of the minute details regarding the response rate of the treatment, all-cause discontinuation, observing the remission rate by any chance, and the baseline and endpoint scores of the depression scale were graded.

The response and remission rates based on the primary outcome scale were provided. To evaluate the data efficacy and acceptability, the corresponding response rate and discontinuation rate were selected as the primary outcome. Secondary outcomes were the remission rate and the endpoint depression score. The patient’s treatment was considered valid if the response rate improvement was ≥50% from the baseline score of the study depression scale, and remission was considered if the remission rate was found to be ≤7, ≤8, or ≤10 on the HAMD-17, HAMD-21, or MADRS, respectively. The discontinuation rate is the percentage of patients who withdrew from the RCT for any reason.

### Risk of bias assessment

The risk of bias was assessed according to the Cochrane Handbook for Systematic Reviews of Interventions (version 5.1.0) and the certainty of evidence. The details for judging the risk of bias were as follows: The investigators clearly described random components in the sequence generation process based on the score criteria of low risk, high risk, and unclear risk. Similarly, other criteria for judging the risk of bias included allocation concealment, blinding of participants and personnel, blinding of outcome assessment, incomplete outcome data, selective reporting, and other bias score criteria based on their low, high, and unclear risks. Two authors (JL and HL) independently evaluated the risk of bias and discussed reaching an agreement.

### Data analysis

STATA version 15.1 was used to perform the pairwise analysis and NMA, while Revman 5.3.3 was used to perform the risk of bias assessment (24). For all data, the appropriate statistical analysis was applied wherever necessary. The relative risk (RR) for dichotomous outcome measures and standardized mean differences for continuous outcomes, combined with a 95% confidence interval (CI), were estimated. In certain cases, if the number of RCTs included was ≥2, pairwise meta-analyses were conducted through a random-effects model to estimate the direct effects of the interventions. Furthermore, statistical heterogeneity was assessed using I^2^ statistics (25–27). If the estimated I^2^ values were <25%, between 25 and 50%, or >50%, the heterogeneity was considered low, moderate, or high, respectively. If I^2^ was ≥50%, subgroup analysis was performed to explore the source of the heterogeneity (27, 28).

Subsequently, NMA was carried out to analyze the mixed effects of direct and indirect comparison based on the frequentist framework using random-effects models (24–26). The network plots were generated for each outcome (i.e., the response rate, remission rate, discontinuation rate, and endpoint depression score) to clarify the direct or indirect comparison of the included studies. Next, the overall rankings for the efficacy and acceptability of each treatment were estimated using the surface under the cumulative ranking curve (SUCRA), which ranged from 0 to 1. The closer the SUCRA value was to 1, the better the treatment effect (24, 29).

A common heterogeneity parameter (similarity, transitivity, and consistency) was assumed for all treatment comparisons. Similarity and transitivity require combining studies (e.g., A vs. C and B vs. C studies) similar to their effect modifiers (study type and population baselines, such as sample size, mean age, and sex) (30). To illustrate similarity, the RCTs of those groups having no significant differences in the population baseline data were included using eligible trials (16). Consistency was evaluated in the loop-specific approach using the inconsistency factor (IF) to compare the direct and indirect treatment effects (14, 31). In addition, the global Wald and the node splitting approach were used to evaluate the heterogeneity of the network (30–32). A *P* < 0.05 for the global Wald or the node splitting test indicated the presence of a statistically significant inconsistency in the network model.

Furthermore, subgroup analyses were conducted to explore the difference in the primary outcomes based on different intervention durations between the two groups (divided into the <4 weeks group and ≥4 weeks group). Also, a funnel plot was made to estimate small-study effects, including comparisons of active intervention and a sham and between active intervention and another active intervention (33). Finally, sensitivity analyses, in which studies with too short (one week) of a treatment duration were excluded, were performed to assess the robustness and reliability of the results.

## Results

**FIGURE 2:**
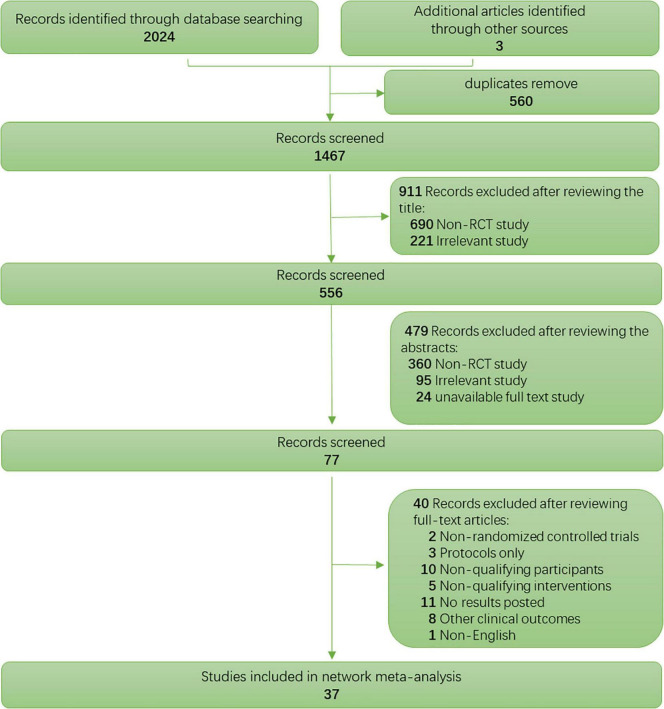
PRISMA flow diagram of the study selection process.

The database search provided 2024 citations, of which only 77 studies were full-text articles retrieved successfully (Figure 2). After thoroughly screening all the publications, only 37 RCTs were finally included, with a publication year between 1999 and 2020. In this NMA (Figure 2), 2120 patients with TRD receiving 10 different strategies of treatments out of 90 treatment arms were included. The main characteristics of all the included studies are reported in Supplementary Table 1. The mean patient age range was 23.5–66.8 years old, and the intervention duration was mainly 2–6 weeks. One study (Miniussi et al.) was carried out for nine weeks, and two studies (Padberg et al. and Baeken et al.) had a duration of one week (34–36). For the RCTs included in this study, there was no variability in the population baselines for the mean age; hence, common transitivity in the NMA study was accepted (Supplementary Figure 3). The characteristics listed for the risk of bias are presented in Supplementary Table 3, with 2, 14, and 21 studies graded as having a high, unclear, and low risk of bias, respectively. Therefore, the two studies (McDonald et al. and Isenberg et al.) with a high risk of bias were excluded from the pairwise and NMA analyses (37, 38).

### Pairwise meta-analysis

A pairwise meta-analysis was conducted for eight groups of inter-comparisons to compare the response rates. First, the sham control was compared with each of the following groups: sham vs. HFL-sup-rTMS (11 RCTs, 200 participants), sham vs. HFL-sub-rTMS (12 RCTs, 213 participants), and sham vs. LFR-HFL-sup-rTMS (10 RCTs, 118 participants). At a later stage of analysis, LFR-sup-rTMS was compared with HFL-sup-rTMS (2 RCTs, 80 participants) and LFR-HFL-sup-rTMS (2 RCTs, 91 participants). Finally, HFL-sup-rTMS (4 RCTs, 80 participants) was compared with LFR-HFL-sup-rTMS, and HFL-sub-rTMS (2 RCTs, 16 participants) was compared with LFL-sub-rTMS.

More efficacious rTMS interventions than the sham control included HFL-sup-rTMS (RR = 4.35, 95% CI: 2.41–7.87, 12 RCTs, 213 participants), HFL-sub-rTMS (RR = 2.24, 95% CI: 1.03–4.87, 12 RCTs, 222 participants), and LFR-HFL-sup-rTMS (RR = 2.36, 95% CI: 1.26–4.40, 10 RCTs, 118 participants). There was no significant difference between the two rTMS groups.

All comparisons were shown to have a low-to-moderate heterogeneity (*I*^2^ < 50%), except for HFL sub-rTMS (*I*^2^ = 68.8%) vs. the sham control. Hence, sensitivity analyses were performed by excluding one of the studies from the HFL-sub-rTMS group vs. the sham control (12 RCTs, 222 participants) at the same time, and the impact of eliminating each of the studies on the overall results between HFL-sub-rTMS and the sham control was estimated.

After the study by Theleritis et al. was excluded, the I^2^ value of HFL-sub-rTMS vs. sham was shown to be 46.9%, with an RR value of 1.72 and a 95% CI of 0.93–3.17 (39). The full text was reviewed, and the risk of bias for the study was reassessed. This study was graded as having an unclear risk of bias. Hence, it was excluded. The detailed information of the sensitivity analyses is reported in Supplementary Table 12, and the direct comparison results, remission rates, endpoint depression scores, and all-cause discontinuation are listed in Supplementary Appendix 4.

### Network meta-analysis

#### Evaluation of statistical heterogeneity and inconsistency

For global heterogeneity, the I^2^ values were 10.18% for efficacy and 6.85% for acceptability. The global Wald test results suggested no statistical inconsistency for the efficacy (*P* = 0.926) or acceptability (*P* = 0.985). For local heterogeneity, the tests of loop inconsistency showed one loop (formed by sham, HFL-sup-rTMS, and LFR-HFL-sup-rTMS) presenting statistical inconsistency for the response (IF = 1.71, 95% CI: 0.58–2.85) and none for acceptability (Supplementary Figure 6). Finally, the node-splitting model alone demonstrated a significant difference between the comparison of HFL-sub-rTMS and LFR-HFL-sup-rTMS in terms of acceptability (*P* = 0.038) but not for efficacy (*P* > 0.05). The details for global and local heterogeneity are documented in the Supplementary Table 9.

#### Relative effects and relative ranking of interventions

##### Efficacy

**FIGURE 3:**
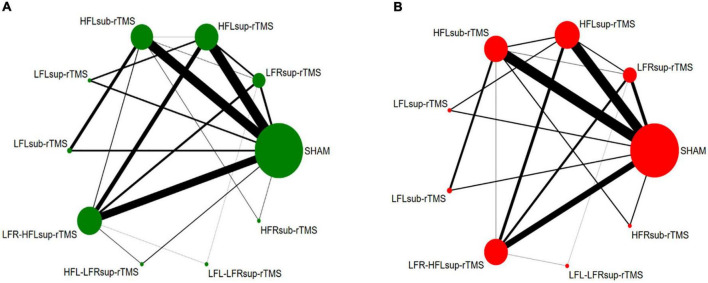
Network plot for efficacy **(A)** and acceptability **(B)**: The width of the lines represents the number of trials comparing each pair of treatments. The size of each node represents the number of randomly assigned participants.

Thirty-five trials (two were removed due to a high risk of bias) reported the response rate and were included in the NMA to compare the efficacy, with a total of 1873 TRD patients. Figure 3A shows the network plot of the efficacy. According to the NMA results, HFL-LFR-sup-rTMS (RR = 5.29, 95% CI: 1.24–22.50), HFL-sup-rTMS (RR = 2.97, 95% CI: 1.74–5.05), LFR-sup-rTMS (RR = 2.72, 95% CI: 1.50–4.90), LFR-HFL-sup-rTMS (RR = 2.71, 95% CI: 1.62–4.53), and HFL-sub-rTMS (RR = 1.91, 95% CI: 1.18–3.10) were more efficacious than the sham control (Table 1). There was no significant difference between the nine active rTMS interventions. According to the ranked orders of the SUCRAs, HFL-LFR-sup-rTMS (84.4%), HFL-sup-rTMS (70.1%), and LFR-HFL-sup-rTMS (63.9%) were the most efficacious.

**TABLE 1:** Comparisons for efficacy and acceptability of treatments.

	LFR-sup-rTMS	HFL-sup-rTMS	HFL-sub-rTMS	LFL-sup-rTMS	LFL-sub-rTMS	LFR-HFL-sup-rTMS	HFL-LFR-sup-rTMS	LFL-LFR-sup-rTMS	HFR-sub-rTMS	sham
LFR-sup-rTMS	— —	0.96	0.73	0.81	0.72	**0.50**	— —	1.03	0.59	0.68
		(0.78, 1.19)	(0.40, 1.35)	(0.03, 22.27)	(0.06, 9.15	**(0.31, 0.80)**		(0.66, 1.61)	(0.02, 17.54)	(0.41, 1.15)
HFL-sup-rTMS	0.92	— —	0.76	0.84	0.74)	**0.52**	— —	1.07	0.62	0.71
	(0.49, 1.71)		(0.41, 1.41)	(0.03, 23.03)	(0.06, 9.50)	**(0.32, 0.84)**		(0.66, 1.74)	(0.02, 18.21)	(0.42, 1.18)
HFL-sub-rTMS	1.42	1.55	— —	1.11	0.98	0.69	— —	1.41	0.81	0.93
	(0.74, 2.74)	(0.84, 2.85)		(0.04, 31.17)	(0.08, 11.84)	(0.37, 1.25)		(0.69, 2.88)	(0.03, 23.28)	(0.57, 1.54)
LFL-sup-rTMS	1.58	1.72	1.11	— —	0.88	0.62	— —	1.27	0.73	0.84
	(0.05, 49.29)	(0.06, 52.09)	(0.04, 34.36)		(0.01, 56.07)	(0.02, 17.20)		(0.05, 35.71)	(0.01, 81.31)	(0.03, 23.03)
LFL-sub-rTMS	1.71	1.87	1.21	1.09	— —	0.70	— —	1.43	0.83	0.95
	(0.12, 23.82)	(0.14, 25.72)	(0.09, 15.70)	(0.02, 77.93)		(0.05, 8.89)		(0.11, 18.79)	(0.01, 53.66)	(0.08, 11.74)
LFR-HFL-sup-rTMS	1.00	1.09	0.70	0.63	0.58	— —	— —	**2.05**	1.18	1.36
	(0.55, 1.81)	(0.59, 2.02)	(0.38, 1.32)	(0.02, 19.70)	(0.04, 8.04)			**(1.19, 3.54)**	(0.04, 34.93)	(0.82, 2.25)
HFL-LFR-sup-rTMS	0.51	0.56	0.36	0.33	0.30	0.51	— —	— —	— —	— —
	(0.11, 2.31)	(0.12, 2.52)	(0.08, 1.63)	(0.01, 13.12)	(0.02, 5.76)	(0.13, 2.10)				
LFL-LFR-sup-rTMS	1.14	1.25	0.80	0.72	0.67	1.14	2.23	— —	0.58	0.66
	(0.46, 2.84)	(0.45, 3.46)	(0.29, 2.26)	(0.02, 24.80)	(0.04, 10.43)	(0.46, 2.84)	(0.42, 11.80)		(0.02, 17.40)	(0.35, 1.25)
HFR-sub-rTMS	3.38	3.69	2.38	2.14	1.97	3.38	6.58	2.96	— —	1.15
	(0.83, 13.71)	(0.93, 14.64)	(0.65, 8.77)	(0.06, 81.91)	(0.11, 34.68)	(0.85, 13.38)	(0.95, 45.63)	(0.59, 14.75)		(0.04, 32.97)
SHAM	**2.72**	**2.97**	**1.91**	1.72	1.59	**2.71**	**5.29**	2.38	0.80	— —
	**(1.50, 4.90)**	**(1.74, 5.05)**	**(1.18, 3.10)**	(0.06, 52.09)	(0.12, 21.04)	**(1.62, 4.53)**	**(1.24, 22.50)**	(0.89, 6.33)	(0.22, 2.94)	

HFR-sub-rTMS, high-frequency rTMS over the right dorsolateral prefrontal cortex at the resting motor sub-threshold; HFL-sub-rTMS, high-frequency rTMS over the left dorsolateral prefrontal cortex at the resting motor sub-threshold; HFL-sup-rTMS, high-frequency rTMS over the left dorsolateral prefrontal cortex at the resting motor sup-threshold; LFL-sup-rTMS, low-frequency rTMS over the left dorsolateral prefrontal cortex at the resting motor sup-threshold; LFL-sub-rTMS, low-frequency rTMS over the left dorsolateral prefrontal cortex at the resting motor sub-threshold; LFR-sup-rTMS, low-frequency rTMS over the right dorsolateral prefrontal cortex at the resting motor sup-threshold; LFR-HFL-sup-rTMS, low frequency over the right followed by high frequency over the left DLPFC at the resting motor sup-threshold; HFL-LFR-sup-rTMS, high frequency over the left followed by low frequency over the right DLPFC at the resting motor sup-threshold; LFL-LFR sup-rTMS, low frequency over the left followed by low frequency over the right DLPFC at the resting motor sup-threshold.

Pooled RR (95% CI) for efficacy and acceptability. The RR of response rates is indicated in the lower triangle, while the RR of acceptability is shown in the upper triangle. RR is considered significant if the 95% CI does not include one, with significant results in bold.

##### Acceptability

Thirty-four trials (two trials with a high risk of bias and one without reporting the discontinuation rate) were included in the NMA to compare the acceptability comprising 1811 TRD patients were included. Figure 3B shows the network plot of the acceptability. NMA demonstrated that LFR-HFL-sup-rTMS was more acceptable than LFR-sup-rTMS (RR = 0.50, 95% CI: 0.31–0.80), HFL-sup-rTMS (RR = 0.52, 95% CI: 0.32–0.84), and LFL-LFR-sup-rTMS (RR = 0.49, 95% CI: 0.28–0.84) (Table 1). Also, there was no significant difference between any treatment measures compared with the sham controls for acceptability (Table 1). The SUCRA results showed that LFL-LFR-sup-rTMS (70.6%), LFR-sup-rTMS (70.0%), and HFL-sup-rTMS (64.9%) were ranked in the three first positions for the response.

##### Remission rates and endpoint scores

Network meta-analysis (NMA) reported that LFR-sup-rTMS and HFL-sup-rTMS allowed participants to achieve remission compared to the sham controls. For HFL-sub-rTMS and HFL-sup-rTMS, endpoint scores were lower and were statistically significant. Similarly, there was no significant difference in the comparison between active treatments for remission rates and endpoint scores (Supplementary Table 8). The SUCRA results both showed that HFL-LFR-sup-rTMS, LFR-sup-rTMS, and LFR-HFL-sup-rTMS were ranked in the three first positions for the remission and endpoint scores. The results of the remission rates and endpoint scores are provided in the Supplementary Figure 5 and Supplementary Tables 8, 9.

### Subgroup analysis

#### Intervention duration of <4 weeks

In this subgroup analysis, 19 trials comprising 723 TRD patients were included, and 9 active treatments were included to compare the efficacy. The results showed that only HFL-sub-rTMS (RR = 2.43, 95% CI: 1.19–4.95) was more efficacious than the sham control. For acceptability, 661 TRD patients from 18 RCTs, including 9 active treatments, were included to assess the acceptability. No treatments revealed significantly lower acceptability than the sham controls. The two active treatments demonstrated no significant difference in efficacy or acceptability (Supplementary Table 10).

#### Intervention duration of ≥4 weeks

In this subgroup analysis, 16 trials that enrolled 1150 patients with TRD were included, with 9 active treatments in 37 treatment arms for efficacy and acceptability. The results suggested that LFR-sup-rTMS (RR = 2.58, 95% CI: 1.08–6.16), HFL-sup-rTMS (RR = 3.17, 95% CI: 1.60–6.31), and LFR-HFL-sup-rTMS (RR = 2.94, 95% CI: 1.54–5.64) were more effective than the sham control. The analysis also showed that LFR-HFL-sup-rTMS was more acceptable than LFR-sup-rTMS (RR = 0.47, 95% CI: 0.29–0.76), HFL-sup-rTMS (RR = 0.48, 95% CI: 0.29–0.80), and LFL-LFR-sup-rTMS (RR = 0.46, 95% CI: 0.26–0.81). Similarly, there was no significant difference between the two active treatments for the efficacy or acceptability (Supplementary Table 11).

### Funnel plot analysis

The two funnel plots for efficacy and acceptability of treatment are depicted in Supplementary Figure 9. Overall, the funnel plots did not suggest any substantial asymmetry; thus, the presence of a publication bias was not suspected.

## Discussion

This systematic review and NMA summarize the comparative effects of 9 rTMS interventions and sham controls based on 37 RCTs, including 2120 patients with TRD. In terms of the response rate, HFL-LFR-sup-rTMS, HFL-sup-rTMS, LFR-sup-rTMS, LFR-HFL-sup-rTMS, and HFL-sub-rTMS were found to be better treatments than the sham controls. Similarly, for remission rates, LFR-sup-rTMS, HFL-sup-rTMS, and LFR-HFL-sup-rTMS were superior to the sham controls (Supplementary Table 8). Also, the evidence in our NMA demonstrated the superiority of HFL-LFR-sup-rTMS followed by HFL-sup-rTMS in all rTMS interventions based on ranked orders of response and remission rates. Additionally, all interventions and sham controls were well accepted, but LFL-LFR-sup-rTMS and LFR-sup-rTMS had the highest likelihood of being the two most acceptable treatments.

Several previous meta-analyses have reported the superiority of bilateral rTMS compared with HF-rTMS, LF-rTMS, and the sham control in response and remission rates (13, 14, 16). This is consistent with the results in the present study that HFL-LFR-sup-rTMS is ranked as the most efficacious treatment in the SUCRA ranking for both response and remission rates. In addition, Grimm et al. have reported that depression is associated with an imbalance in bilateral DLPFC activity. Moreover, other studies have reported a reduced left DLPFC and increased metabolism at the right DLPFC (40).

Researchers also have illustrated the excitatory effects of HF stimulation (>5 Hz) and the inhibitory effects of LF stimulation (<1 Hz) in the brain. The excitatory or inhibitory effects may be that rTMS can alter synaptic plasticity, mainly the long-term potentiation/depression of excitatory synaptic transmission (22, 41). Altogether, these findings provide the theoretical basis for rTMS in treating MDD. However, when comparing bilateral rTMS and other unilateral rTMS, no significant differences were found in the RR for response and remission rates or the SMD for endpoint scores. Therefore, additional large head-to-head RCTs comparing bilateral rTMS and unilateral rTMS in the treatment of TRD are needed.

In addition, a further division of the order of stimulus position in bilateral rTMS was made in the present study (i.e., HFL-LFR-sup-rTMS and LFR-HFL-sup-rTMS). HFL-LFR-sup-rTMS was found to be the most likely efficacious measure. LFR-HFL-sup-rTMS was ranked in the third position for both response and remission. Although some research reports have revealed an insignificant difference for HFL-LFR-sup-rTMS or LFR-HFL-sup-rTMS in a sham-controlled study, there was a trend for subjects in the HFL-LFR-sup-rTMS group to show improvement in the HAMD (42). To date, no studies have directly compared the effect of the order of stimulus positions in the treatment of TRD using rTMS. The present study is the first to provide evidence that HFL-LFR-sup-rTMS may have a better efficacy than LFR-HFL-sup-rTMS. However, these results should be interpreted carefully, since there is only one RCT focusing on HFL-LFR-sup-rTMS at present. Further studies should be conducted to clarify the effects of the order of stimulus positions in rTMS (16).

Additionally, our study found that selection of RMT > 100% was more effective and tolerable than RMT ≤ 100%. RMT was defined as the minimum intensity that produces a response in either the abductor pollicis brevis or the first dorsal interosseous muscle for ≥50% of the stimuli (15, 42). The intensity is generally indicated as a percentage of the RMT (15, 42). However, several studies have demonstrated a dose–response relationship between stimulus intensity and efficacy (43). A significant treatment effect was not found when using sub-rTMS for TRD, compared to the sham control, which supports the use of suprathreshold stimulation (RMT > 100%) in rTMS treatment. However, others have argued that there is not a purely linear relationship between the intensity and efficacy. One study observed a change in intensity during rTMS treatment, affecting the severity of depressive and anxiety symptoms after treatment (44). Hence, further studies should pay more attention to this aspect.

Finally, subgroup analyses were performed based on different intervention durations (<4 weeks and ≥4 weeks). We found a better performance for rTMS than sham controls after 4 weeks, expounding the relationship between efficacy and intervention duration, which is consistent to some extent with the results of previous studies. Some studies have reported more dramatic treatment effects as early as 6 weeks in the acute phase (7). The time commitment required for a 4–6-week treatment course may need to be made. Because the intervention time for the RCTs included in our study was between 1 week and 9 weeks, the trend in the efficacy of a treatment course >4 weeks is still inconclusive (15, 45).

### Limitations

Our study has some limitations that must be addressed. First, there was uncertainty in the estimations of the treatment outcomes. A few direct comparisons between two active interventions were included, but 15 of 35 did not report the remission rate. In addition, through 37 RCTs (2 RCTs were not analyzed due to a high risk of bias), 15 of 37 interventions were combined with more than two treatment arms, including 2120 patients.

Second, small-study effects possibly introduced bias as 65% of the trials included fewer than 50 participants. Also, because depressive symptoms in TRD patients were evaluated through either HAMD or MARDS, some methodological heterogeneity might exist. Therefore, the HAMD score followed by the MADRS score was used to calculate the response and remission rates; four studies using only MADRS as the assessment measure were included. Besides, approximately 30% of the studies had an unclear risk of bias, and two studies with a high risk of bias were removed from the analysis. Most of the trials presented an unclear randomization and assignment bias risk, leading to uncertainty for estimates of treatment efficacy.

Additionally, bipolar disorder is a potential cause of TRD (14). Of the included studies, only one trial included patients with bipolar TRD. Thus, subgroup analysis was not conducted to compare the efficacy of rTMS treatment between bipolar and unipolar depression. More trials regarding rTMS treatment of bipolar depression are warranted to gather more evidence. Also, we only used the all-cause discontinuation to assess the acceptability in the present study. However, the higher acceptability may result from efficacy instead of tolerability, leading to an overestimation of tolerability. Therefore, dropout due to the side effects needs to be investigated in future studies.

Another important shortcoming is the failure to assess the degree of cognitive improvement. Cognitive impairment is an important clinical feature of TRD and indicates a poor response to medication. However, only a few of the 37 RCTs conducted pre- and post-treatment cognitive assessments, resulting in insufficient data to allow meta-analysis. Moreover, the treatment duration of two RCTs was only one week. Therefore, sensitivity analysis was performed to assess the robustness and reliability of the results by removing these two trials. The sensitivity analysis results were not affected by these two trials (Supplementary Table 13).

Finally, although we used SUCRA to estimate the ranking order of the comparative effectiveness, the results need to be interpreted with caution because this method may only provide supportive but not conclusive evidence for treatment options (46, 47).

## Conclusion

This NMA explored the clinical efficacy and acceptability between rTMS modalities. The results suggest that HFL-LFR-sup-rTMS, HFL-sup-rTMS, LFR-sup-rTMS, LFR-HFL-sup-rTMS, and HFL-sup-rTMS are more effective than the sham controls. HFL-LFR-sup-rTMS and HFL-sup-rTMS may be the best among the most efficacious rTMS treatments. All interventions and shams were well accepted, while LFL-LFR-sup-rTMS had the highest likelihood of being the most acceptable treatment. The results also demonstrate the relationship between efficacy and intervention duration and suggest a treatment course of more than four weeks.

## Data availability statement

The original contributions presented in this study are included in the article/Supplementary material, further inquiries can be directed to the corresponding author.

## Author contributions

JL, HL, and LC: conception and design of the study. JL and HL: acquisition of data. JL and LC: analysis and interpretation of data. JL: drafting the manuscript. HL and LC: revising the manuscript. All authors reviewed and approved the final version of this manuscript.
