# Supplementary material for: Optimal parameter determination of repetitive transcranial magnetic stimulation for treating treatment-resistant depression: A network meta-analysis of randomized controlled trials
Source: Front Psychiatry. 2022 Dec 1;13:1038312. doi: 10.3389/fpsyt.2022.1038312 (PMC9751374; doi:10.3389/fpsyt.2022.1038312)
Supplement: Supplementary file 1 [file Data_Sheet_1.docx]

Supplementary Material

Appendix 1. Search strategy

Table 1. Search strategy------------------------------------------------------------------------3

Appendix 2. Characteristics of the 37 studies in the meta-analysis

Table 2：Characteristics of the 37 studies---------------------------------------------------4-9

Appendix 3. Risk of bias assessment

Figure 1：Results of risk of bias--------------------------------------------------------------10

Table 3：Grading and support basis of bias risk--------------------------------------------11-15

Appendix 4: Results from pairwise meta-analyses

Figure 2：Forest plot of response rates-------------------------------------------------------16-17

Table 4: Summary of pairwise meta-analyses for the response rate-----------------------18-20

Table 5: Summary of pairwise meta-analyses for the discontinuation rate---------------21-23

Table 6: Summary of pairwise meta-analyses for the remission rate----------------------24-25

Table 7: Summary of pairwise meta-analyses for the endpoint depression score -------26-28

Appendix 5: Assessment of transitivity

Figure 3: Assessment of transitivity------------------------------------------------------------29

Appendix 6: Results from network meta-analyses

Figure 4: Network plot of the remission rate and the endpoint depression score--------30

Table 8: Treatment comparisons of remission rates and endpoint-scores-----------------31

Figure 5: Plots of surface under the cumulative ranking curve (SUCRA) results--------32

Table 9: SUCRA results for each treatment---------------------------------------------------33

Appendix 7: Assessment of consistency

Figure6: Loop inconsistency---------------------------------------------------------------------34

Table9: Test of heterogeneity in the global Wald test and the node splitting approach—35-36

Appendix 8: Results of subgroup analysis

Table 10: Comparisons of the efficacy and acceptability for the subgroup＜4weeks--37-38

Table 11: Comparisons of the efficacy and acceptability for the subgroup ≥4weeks-39

Appendix 9: Sensitivity analysis

Table 12: Results of sensitivity analysis in pair meta-analyses----------------------------40

Figure 7: Network plot of sensitivity analyses for the efficacy and acceptability in network meta-analyses--------------------------------------------------------------------------------------------41

Table 13: Comparisons for efficacy and acceptability in sensitivity analyses in network meta-analyses--------------------------------------------------------------------------------------------41-42

Figure 8: Plots of surface under the cumulative ranking curve (SUCRA) results in sensitivity analyses--------------------------------------------------------------------------------------------43

Table 14: SUCRA values for the efficacy and acceptability in sensitivity analyses----44

Appendix 10: Funnel plot for the efficacy and acceptability of the included treatments

Figure 9: Funnel plot for the efficacy of the included treatments and the acceptability of the included treatments-------------------------------------------------------------------------------45

Appendix 11: References of included studies---------------------------------------------------46-50

**Appendix 1. Search strategy**

**Table 1. Search strategy**

|  | Subject-words | free-text | free-text |
| --- | --- | --- | --- |
| #1 | Transcranial Magnetic Stimulation | Magnetic Stimulation, Transcranial | Transcranial Magnetic Stimulations |
|  |  | Magnetic Stimulations, Transcranial | Transcranial Magnetic Stimulation, Single Pulse |
|  |  | Stimulation, Transcranial Magnetic | Transcranial Magnetic Stimulation, Paired Pulse |
|  |  | Stimulations, Transcranial Magnetic | Transcranial Magnetic Stimulation, Repetitive |
| #2 | Depressive Disorder, Treatment-Resistant | Depressive Disorder, Treatment Resistant | Depressions, Refractory |
|  |  | Depressive Disorders, Treatment-Resistant | Refractory Depressions |
|  |  | Disorder, Treatment-Resistant Depressive | Therapy-Resistant Depression |
|  |  | Disorders, Treatment-Resistant Depressive | Depression, Therapy-Resistant |
|  |  | Treatment-Resistant Depressive Disorder | Depressions, Therapy-Resistant |
|  |  | Treatment-Resistant Depressive Disorders | Therapy Resistant Depression |
|  |  | Depressive Disorder, Treatment Resistant | Therapy-Resistant Depressions |
|  |  | Depressive Disorders, Treatment-Resistant | Treatment Resistant Depression |
|  |  | Disorder, Treatment-Resistant Depressive | Depression, Treatment Resistant |
|  |  | Disorders, Treatment-Resistant Depressive | Depressions, Treatment Resistant |
|  |  | Refractory Depression | Resistant Depression, Treatment |
|  |  | Depression, Refractory | Resistant Depressions, Treatment |

**Appendix 2. Characteristics of the 37 studies in the meta-analysis.**

| **Source** | **Region** | **Cross-over** | **Sample size（Male/Female)** | **Failed trials（minimum/average)** | **Mean Age** | | | | **With bipolar depression** | **With psychotic symptoms** | **Intervention methods**  **Table 2：Characteristics of the 37 studies** | | | | **Intervention Duration** | **with antidepressent drugs** | **Evaluation scale** | **baseline score** | | | | **Response** | | | | **Remission** | | | | **post-treatment score** | | | | **all-cause discontinuation** | | | |
| --- | --- | --- | --- | --- | --- | --- | --- | --- | --- | --- | --- | --- | --- | --- | --- | --- | --- | --- | --- | --- | --- | --- | --- | --- | --- | --- | --- | --- | --- | --- | --- | --- | --- | --- | --- | --- | --- |
|  |  |  |  |  | **group1** | **group2** | **group3** | **group4** |  |  |  |  |  |  |  |  |  |  |  |  |  |  |  |  |  |  |  |  |  |  |  |  |  |  |  |  |  |
|  |  |  |  |  |  |  |  |  |  |  | **group1** | **group2** | **group3** | **group4** |  |  |  | **group1** | **group2** | **group3** | **group4** | **group1** | **group2** | **group3** | **group4** | **group1** | **group2** | **group3** | **group4** | **group1** | **group2** | **Group3** | **Group4** | **group1** | **group2** | **Group3** | **Group4** |
|  |  |  |  |  |  |  |  |  |  |  |  |  |  |  |  |  |  |  |  |  |  |  |  |  |  |  |  |  |  |  |  |  |  |  |  |  |  |
| **Paul B. Fitzgerald** | **Australia** | **No** | **300(87/213)** | **≥2/N.A.** | **46.6±12.7** | **44.4±11.3** | **49.3±16.8** | **46.2±14.5** | **Yes** | **Yes** | **HFL sup-rTMS，2250 pulses per session** | **HFL sup-rTMS，5625 pulses per session** | **LFR sup-rTMS，1200 pulses per session** | **LFR sup-rTMS，3600 pulses per session** | **4 weeks** | **Yes** | **HRSD-17** | **26.9 ±6.0** | **26.1±6.7** | **25.8±6.3** | **25.7±5.8** | **52.5%（31/59）** | **47.3%（43/91）** | **49.1%（28/57）** | **48.4%（45/93）** | **18.6%（11/59）** | **31.9%（29/91）** | **26.3%（15/57）** | **33.3%（31/93）** | **12** | **13** | **13** | **11** | **26/59** | **51/91** | **15/57** | **63/93** |
|  |  |  |  |  |  |  |  |  |  |  |  |  |  |  |  |  |  |  |  |  |  |  |  |  |  |  |  |  |  |  |  |  |  |  |  |  |  |
| **2019** |  |  |  |  |  |  |  |  |  |  | **n=59** | **n=91** | **n=57** | **n=93** |  |  |  |  |  |  |  |  |  |  |  |  |  |  |  |  |  |  |  |  |  |  |  |
| **Paul B. Fitzgerald** | **Australia** | **Yes** | **27(15/12)** | **≥2/N.A.** | **42.12±9.32** | **46.54±11.43** | **N.A.** | **N.A.** | **No report** | **yes** | **HFL sub-rTMS** | **LFR sup-rTMS** | **N.A.** | **N.A.** | **4 weeks** | **Yes** | **MADRS** | **33.68±3.97** | **34.27±4.98** | **N.A.** | **N.A.** | **44%（7/16）** | **45%（5/11）** | **N.A.** | **N.A.** | **19%（3/16）** | **36%（4/11）** | **N.A.** | **N.A.** | **18.31±11.48** | **19.90±14.52** | **N.A.** | **N.A.** | **3/16** | **5/11** | **N.A.** | **N.A.** |
|  |  |  |  |  |  |  |  |  |  |  |  |  |  |  |  |  |  |  |  |  |  |  |  |  |  |  |  |  |  |  |  |  |  |  |  |  |  |
| **2007** |  |  |  |  |  |  |  |  |  |  | **n=16** | **n=11** |  |  |  |  |  |  |  |  |  |  |  |  |  |  |  |  |  |  |  |  |  |  |  |  |  |
| **Alisson Paulino Trevizol** | **the United states** | **No** | **43(20/23)** | **2/N.A.** | **66.8±5.8** | **66.1±8.5** | **64.1±3.7** | **N.A.** | **No report** | **No** | **LFR-HFL sup-rTMS， 2100 pulses per session** | **HFL sup-rTMS， 2100 pulses per session** | **sham-rTMS** | **N.A.** | **6 weeks** | **Yes** | **HRSD-17** | **24.6±4.2** | **26.5±3.4** | **24.5±3.5** | **N.A.** | **45%（9/20）** | **0（0/11）** | **16.7%（2/12）** | **N.A.** | **40%(8/20)** | **0(0/11)** | **0(0/12)** | **N.A.** | **N.A.** | **N.A.** | **N.A.** | **N.A.** | **1/20** | **2/11** | **1/12** | **N.A.** |
| **2019** |  |  |  |  |  |  |  |  |  |  | **n=20** | **n=11** | **n=12** |  |  |  |  |  |  |  |  |  |  |  |  |  |  |  |  |  |  |  |  |  |  |  |  |
| **Paul B. Fitzgerald** | **Australia** | **No** | **50(19/31)** | **≥2/5.9±3.0** | **46.8±10.7** | **43.7± 10.2** | **N.A.** | **N.A.** | **Yes** | **No** | **LFR-HFL sup-rTMS** | **Sham-rTMS** | **N.A.** | **N.A.** | **6 weeks** | **Yes** | **MADRS** | **34.0±5.9** | **34.1± 5.2** | **N.A.** | **N.A.** | **(44%)11/25** | **(8%)2/25** | **N.A.** | **N.A.** | **(36%)9/25** | **0/25** | **N.A.** | **N.A.** | **N.A.** | **N.A.** | **N.A.** | **N.A.** | **0/25** | **3/25** | **N.A.** | **N.A.** |
| **2006** |  |  |  |  |  |  |  |  |  |  |  |  |  |  |  |  |  |  |  |  |  |  |  |  |  |  |  |  |  |  |  |  |  |  |  |  |  |
|  |  |  |  |  |  |  |  |  |  |  | **n=25** | **n=25** |  |  |  |  |  |  |  |  |  |  |  |  |  |  |  |  |  |  |  |  |  |  |  |  |  |
| **William M. McDonald** | **the United states** | **No** | **62(30/32)** | **≥3/N.A.** | **54.0 (47.0, 64.0)** | **49.0 (41.0, 55.0)** | **49.0 (39.0, 54.0)** | **N.A.** | **Yes** | **Yes** | **Sham -rTMS** | **HFL-LFR sup-rTMS,1600 pulses per session** | **LFR-HFL sup-rTMS,1600 pulses per session** | **N.A.** | **2 weeks** | **Yes** | **HDRS-17** | **N.A.** | **N.A.** | **N.A.** | **N.A.** | **8%(1/12)** | **28%(7/25)** | **12%(3/25)** | **N.A.** | **0/12** | **3/25** | **0/25** | **N.A.** | **N.A.** | **N.A.** | **N.A.** | **N.A.** | **N.A.** | **N.A.** | **N.A.** | **N.A.** |
| **2006** |  |  |  |  |  |  |  |  |  |  |  |  |  |  |  |  |  |  |  |  |  |  |  |  |  |  |  |  |  |  |  |  |  |  |  |  |  |
|  |  |  |  |  |  |  |  |  |  |  | **n=12** | **n=25** | **n=25** |  |  |  |  |  |  |  |  |  |  |  |  |  |  |  |  |  |  |  |  |  |  |  |  |
| **Paul B. Fitzgerald** | **Australia** | **No** | **219(74/148)** | **≥2/5.40±2.8** | **45.68 ±13.7** | **47.91± 13.7** | **47.93± 14.1** | **N.A.** | **Yes** | **No report** | **LFR-HFL sup-rTMS,1800 pulses per session** | **LFL-LFR sup-rTMS,1800 pulses per session** | **LFR sup-rTMS,900 pulses per session** | **N.A.** | **4 weeks** | **Yes** | **HAMD-17** | **21.2±5.6** | **20.9±5.3** | **21.8±4.7** | **N.A.** | **56.3%（40/71）** | **48.7%（37/76）** | **54.9%（39/71）** | **N.A.** | **35.20%** | **28.90%** | **31.00%** | **N.A.** | **9.93± 5.94** | **9.22± 5.27** | **9.02±4.86** | **N.A.** | **11/71** | **25/76** | **23/71** | **N.A.** |
| **2011** |  |  |  |  |  |  |  |  |  |  |  |  |  |  |  |  |  |  |  |  |  |  |  |  |  |  |  |  |  |  |  |  |  |  |  |  |  |
|  |  |  |  |  |  |  |  |  |  |  | **n=71** | **n=76** | **n=71** |  |  |  |  |  |  |  |  |  |  |  |  |  |  |  |  |  |  |  |  |  |  |  |  |
| **S. PALLANTI** | **the United states** | **No** | **60(25/35)** | **≥2/5.91±1.74** | **47.60± 12.33** | **51.20± 12.53** | **47.85± 9.12** | **N.A.** | **NO** | **Yes** | **LFR-HFL sup-rTMS,1420 pulses per session** | **LFR-HFL sup-rTMS,1420 pulses per session** | **sham-rTMS** | **N.A.** | **3 weeks** | **Yes** | **HAMD-17** | **28.75± 6.01** | **27.95± 5.89** | **29.05±3.54** | **N.A.** | **20%（4/20）** | **35%（7/20）** | **10%（2/20）** | **N.A.** | **10%（2/20）** | **30（6/20）** | **5%（1/20）** | **N.A.** | **N.A.** | **N.A.** | **N.A.** | **N.A.** | **0/20** | **0/20** | **0/20** | **N.A.** |
| **2010** |  |  |  |  |  |  |  |  |  |  |  |  |  |  |  |  |  |  |  |  |  |  |  |  |  |  |  |  |  |  |  |  |  |  |  |  |  |
|  |  |  |  |  |  |  |  |  |  |  | **n=20** | **n=20** | **n=20** |  |  |  |  |  |  |  |  |  |  |  |  |  |  |  |  |  |  |  |  |  |  |  |  |
| **DANIEL M. BLUMBERGER** | **Canada** | **No** | **68(28/40)** | **≥2/N.A.** | **58.0±12.5** | **48.9±13.4** | **45.8±13.4** | **N.A.** | **No report** | **NO** | **LFR-HFL sup-rTMS , 1215 pulses per session** | **HFL sub-rTMS, 1450 pulses per session** | **sham-rTMS** | **N.A.** | **6 weeks** | **Yes** | **HDRS-17** | **25.1±3.8** | **26.0±3.3** | **25.2±2.8** | **N.A.** | **38.5%(10/26)** | **4.5%(1/22)** | **10.0%(2/20)** | **N.A.** | **34.6%（9/26）** | **4.5%（1/22）** | **5.0%（1/20）** | **N.A.** | **14.4±8.3** | **20.3±5.1** | **18.9±6.4** | **N.A.** | **4/26** | **10/22** | **5/20** | **N.A.** |
| **2012** |  |  |  |  |  |  |  |  |  |  | **n=26** | **n=22** | **n=20** |  |  |  |  |  |  |  |  |  |  |  |  |  |  |  |  |  |  |  |  |  |  |  |  |
| **Andrew M. Speer** | **the United states** | **No** | **24(13/11)** | **≥2/N.A.** | **39.6±9.0** | **41.3±14.5** | **44.9±9.1** | **N.A.** | **Yes** | **No report** | **LFL sup-rTMS,1600 pulses per session** | **HFL sup-rTMS,1600 pulses per session** | **sham** | **N.A.** | **3 weeks** | **No** | **HAMD-28** | **28.6±7.6** | **35.8±10.6** | **24.0±4.6** | **N.A.** | **0/8** | **0/8** | **0/8** | **N.A.** | **0/8** | **0/8** | **0/8** | **N.A.** | **25.1± 6.9** | **32.5± 10.1** | **29.3±6.0** | **N.A.** | **0/8** | **0/8** | **0/8** | **N.A.** |
| **2014** |  |  |  |  |  |  |  |  |  |  |  |  |  |  |  |  |  |  |  |  |  |  |  |  |  |  |  |  |  |  |  |  |  |  |  |  |  |
|  |  |  |  |  |  |  |  |  |  |  | **n=8** | **n=8** | **n=8** |  |  |  |  |  |  |  |  |  |  |  |  |  |  |  |  |  |  |  |  |  |  |  |  |
| **Minna Valkonen-Korhonen** | **Finland** | **No** | **37(22/15)** | **≥2/N.A.** | **37.1±11.1** | **36.4±15.3** | **N.A.** | **N.A.** | **No** | **Yes** | **LFR-HFL sup-rTMS** | **sham-rTMS** | **N.A.** | **N.A.** | **6 weeks** | **Yes** | **HAMD-17** | **27.7±6.9** | **25.9±5.4** | **N.A.** | **N.A.** | **44.4%(8/18)** | **57.9%(11/19)** | **N.A.** | **N.A.** | **22.2%(4/18)** | **31.6%(6/19)** | **N.A.** | **N.A.** | **12.7±7.1** | **12.8±7.1** | **N.A.** | **N.A.** | **2/18** | **1/19** | **N.A.** | **N.A.** |
|  |  |  |  |  |  |  |  |  |  |  |  |  |  |  |  |  |  |  |  |  |  |  |  |  |  |  |  |  |  |  |  |  |  |  |  |  |  |
| **2018** |  |  |  |  |  |  |  |  |  |  | **n=18** | **n=19** |  |  |  |  |  |  |  |  |  |  |  |  |  |  |  |  |  |  |  |  |  |  |  |  |  |
| **Paul B. Fitzgerald** | **Australia** | **Yes** | **59(31/28)** | **≥2/ 8.9 ± 16.9** | **50.3±13.7** | **42.6± 13.6** | **46.4±15.3** | **N.A.** | **No** | **Yes** | **HFL sup-rTMS，1200 pulses per session** | **LFR sup-rTMS，600 pulses per session** | **LFR-HFL sup-rTMS，1800 pulses per session** | **N.A.** | **3 weeks** | **Yes** | **MADRS** | **34.2±5.6** | **32.4±6.4** | **33.3±7.3** | **N.A.** | **0/21** | **0/18** | **0/20** | **N.A.** | **N.A.** | **N.A.** | **N.A.** | **N.A.** | **N.A.** | **33.4±8.7** | **30.4±6.7** | **30.2±11.2** | **0/21** | **1/18** | **0/20** | **N.A.** |
|  |  |  |  |  |  |  |  |  |  |  | **n=21** | **n=18** | **n=20** | **N.A.** |  |  |  |  |  |  |  |  |  |  |  |  |  |  |  |  |  |  |  |  |  |  |  |
|  |  |  |  |  |  |  |  |  |  |  |  |  |  |  |  |  |  |  |  |  |  |  |  |  |  |  |  |  |  |  |  |  |  |  |  |  |  |
| **2018** |  |  |  |  |  |  |  |  |  |  |  |  |  |  |  |  |  |  |  |  |  |  |  |  |  |  |  |  |  |  |  |  |  |  |  |  |  |
| **C. Miniussi** | **Italy** | **No** | **20(5/15)** | **≥5/N.A.** | **52** | **58** | **N.A.** | **N.A.** | **Yes** | **Yes** | **LFL sub-rTMS，2000 pulses per session** | **HFL sub-rTMS，2040 pulses per session** | **N.A.** | **N.A.** | **9weeks** | **Yes** | **HDRS-21** | **22.70± 7.20** | **20.50±3.75** | **N.A.** | **N.A.** | **0/10** | **0/10** | **N.A.** | **N.A.** | **N.A.** | **N.A.** | **N.A.** | **N.A.** | **10.21±4.58** | **14.64±5.58** | **N.A.** | **N.A.** | **0/10** | **0/10** | **N.A.** | **N.A.** |
|  |  |  |  |  |  |  |  |  |  |  |  |  |  |  |  |  |  |  |  |  |  |  |  |  |  |  |  |  |  |  |  |  |  |  |  |  |  |
| **2005** |  |  |  |  |  |  |  |  |  |  | **n=10** | **n=10** |  |  |  |  |  |  |  |  |  |  |  |  |  |  |  |  |  |  |  |  |  |  |  |  |  |
| **Frank Padberg** | **Germany** | **No** | **18(7/11)** | **≥2/3.4±1.5** | **63.5±15.8** | **46.7±14.7** | **43.3±11.6** | **N.A.** | **No** | **No report** | **HFL sub-rTMS ，250 pulses per session** | **LFL sub-rTMS，250 pulses per session** | **sham-rTMS** | **N.A.** | **1 week** | **Yes** | **HAMD-21** | **30.2±9.5** | **26.7±9.4** | **22.2±8.8** | **N.A.** | **0/6** | **0/6** | **0/6** | **N.A.** | **0/6** | **0/6** | **0/6** | **N.A.** | **28.5±9.4** | **21.5±21.5** | **23.5±10.4** | **N.A.** | **0/6** | **0/6** | **0/6** | **N.A.** |
| **1999** |  |  |  |  |  |  |  |  |  |  | **n=6** | **n=6** | **n=6** |  |  |  |  |  |  |  |  |  |  |  |  |  |  |  |  |  |  |  |  |  |  |  |  |
| **Daniel M. Blumberger** | **Canada** | **No** | **121(44/77）** | **≥2/7.4±5.9** | **46.4 ± 12.5** | **46.5 ± 14.1** | **48.1 ± 12.0** | **N.A.** | **No** | **No report** | **LFR-HFL sup-rTMS,2100 pulses per session** | **HFL sup-rTMS,2100 pulses per session** | **sham-rTMS** | **N.A.** | **3 or 6 weeks** | **Yes** | **HAMD-17** | **24.1±3.2** | **26±3.4** | **25.5±3.6** | **N.A.** | **9/40** | **6/40** | **2/41** | **N.A.** | **8/40** | **3/40** | **1/41** | **N.A.** | **N.A.** | **N.A.** | **N.A.** | **N.A.** | **4/40** | **7/40** | **5/41** | **N.A.** |
| **2016** |  |  |  |  |  |  |  |  |  |  | **n=40** | **n=40** | **n=41** |  |  |  |  |  |  |  |  |  |  |  |  |  |  |  |  |  |  |  |  |  |  |  |  |
| **Mauro Garcia-Toro** | **Spain** | **N.A.** | **20(9/11)** | **≥2 / N.A.** | **48.50±13.28** | **47.20±11.8** | **N.A.** | **N.A.** | **No** | **No** | **LFR-HFL sup-rTMS** | **sham-rTMS** | **N.A.** | **N.A.** | **2 weeks** | **Yes** | **HAMD-21** | **27.30±4.97** | **25.10±7.28** | **N.A.** | **N.A.** | **20%（2/10）** | **0/10** | **N.A.** | **N.A.** | **N.A.** | **N.A.** | **N.A.** | **N.A.** | **20.10±8.18** | **23.60±7.79** | **N.A.** | **N.A.** | **0/10** | **0/10** | **N.A.** | **N.A.** |
| **2005** |  |  |  |  |  |  |  |  |  |  | **n=10** | **n=10** |  |  |  |  |  |  |  |  |  |  |  |  |  |  |  |  |  |  |  |  |  |  |  |  |  |
| **David H. Avery** | **the United states** | **No** | **68(31/37)** | **≥2/N.A.** | **23.5±3.9** | **25.4± 11.7** | **N.A.** | **N.A.** | **No** | **No** | **HFL sup-rTMS,1600 pulses per session** | **sham-rTMS** | **N.A.** | **N.A.** | **4 weeks** | **Yes** | **HDRS-17** | **23.5±3.9** | **23.5±2.9** | **N.A.** | **N.A.** | **11/35** | **2/33** | **N.A.** | **N.A.** | **7/35** | **1/33** | **N.A.** | **N.A.** | **N.A.** | **N.A.** | **N.A.** | **N.A.** | **3/35** | **3/35** | **N.A.** | **N.A.** |
|  |  |  |  |  |  |  |  |  |  |  |  |  |  |  |  |  |  |  |  |  |  |  |  |  |  |  |  |  |  |  |  |  |  |  |  |  |  |
| **2005** |  |  |  |  |  |  |  |  |  |  | **n=35** | **n=33** |  |  |  |  |  |  |  |  |  |  |  |  |  |  |  |  |  |  |  |  |  |  |  |  |  |
| **Paul B. Fitzgerald** | **Australia** | **No** | **66(29/37)** | **≥2/5.20±3.3** | **40.45± 15.5** | **43.4±12.7** | **44.9±15.7** | **N.A.** | **No** | **No** | **LFR-HFL sup-rTMS** | **HFL sup-rTMS** | **sham-rTMS** | **N.A.** | **3 weeks** | **Yes** | **HAMD-17** | **24.3±3.6** | **23.7±3.8** | **22.9±2.1** | **N.A.** | **1/22** | **0/24** | **0/20** | **N.A.** | **N.A.** | **N.A.** | **N.A.** | **N.A.** | **22.2±6.0** | **19.6±4.2** | **22.6±5.0** | **N.A.** | **3/22** | **0/24** | **3/17** | **N.A.** |
|  |  |  |  |  |  |  |  |  |  |  |  |  |  |  |  |  |  |  |  |  |  |  |  |  |  |  |  |  |  |  |  |  |  |  |  |  |  |
| **2012** |  |  |  |  |  |  |  |  |  |  | **n=22** | **n=24** | **n=20** |  |  |  |  |  |  |  |  |  |  |  |  |  |  |  |  |  |  |  |  |  |  |  |  |
| **Paul B. Fitzgerald** | **Australia** | **No** | **46(20/26)** | **≥2/N.A.** | **46.3± 12.6** | **49.7±1 1.0** | **N.A.** | **N.A.** | **Only** | **No report** | **LFR-HFL sup-rTMS,2000 pulses per session** | **Sham-rTMS** | **N.A.** | **N.A.** | **4 weeks** | **Yes** | **HAMD-17** | **23.2±4.0** | **23.0±5.1** | **N.A.** | **N.A.** | **3/23** | **1/23** | **N.A.** | **N.A.** | **2/23** | **0/23** | **N.A.** | **N.A.** | **19.8±5.7** | **20.0±4.8** | **N.A.** | **N.A.** | **4/23** | **2/23** | **N.A.** | **N.A.** |
|  |  |  |  |  |  |  |  |  |  |  |  |  |  |  |  |  |  |  |  |  |  |  |  |  |  |  |  |  |  |  |  |  |  |  |  |  |  |
| **2016** |  |  |  |  |  |  |  |  |  |  | **n=23** | **n=23** |  |  |  |  |  |  |  |  |  |  |  |  |  |  |  |  |  |  |  |  |  |  |  |  |  |
| **Huirong Zheng** | **China** | **No** | **32(20/12)** | **≥2/N.A.** | **26.9±6.4** | **26.9±4.3** | **N.A.** | **N.A.** | **Yes** | **No report** | **HFL sup-rTMS,3000 pulses per session** | **Sham-rTMS** | **N.A.** | **N.A.** | **4 weeks** | **Yes** | **HAMD-17** | **23.1±3.6** | **23.6±3.6** | **N.A.** | **N.A.** | **11/18** | **1/14** | **N.A.** | **N.A.** | **N.A.** | **N.A.** | **N.A.** | **N.A.** | **13.5±5.1** | **22.9±3.4** | **N.A.** | **N.A.** | **0/18** | **0/14** | **N.A.** | **N.A.** |
| **2015** |  |  |  |  |  |  |  |  |  |  | **n=18** | **n=14** |  |  |  |  |  |  |  |  |  |  |  |  |  |  |  |  |  |  |  |  |  |  |  |  |  |
| **Hongjun Peng** | **China** | **No** | **30(19/11)** | **≥2/N.A.** | **27.4 ±6.1** | **26.4±3.5** | **N.A.** | **N.A.** | **Yes** | **No report** | **HFL sup-rTMS,3000 pulses per session** | **Sham-rTMS** | **N.A.** | **N.A.** | **4 weeks** | **Yes** | **HAMD-17** | **24.7 ±3.1** | **24.5 ±3.3** | **N.A.** | **N.A.** | **10/17** | **1/13** | **N.A.** | **N.A.** | **N.A.** | **N.A.** | **N.A.** | **N.A.** | **13.5±5.1** | **22.9±3.4** | **N.A.** | **N.A.** | **0/17** | **0/13** | **N.A.** | **N.A.** |
| **2012** |  |  |  |  |  |  |  |  |  |  | **n=17** | **n=13** |  |  |  |  |  |  |  |  |  |  |  |  |  |  |  |  |  |  |  |  |  |  |  |  |  |
| **Tung-Ping Su** | **China** | **No** | **33(8/22)** | **≥2/N.A.** | **43.6± 12.0** | **43.2± 10.6** | **42.6 ±11.0** | **N.A.** | **Yes** | **No** | **HFL sub-rTMS,1600 pulses per session** | **HFL sub-rTMS,1600 pulses per session** | **SHM** | **N.A.** | **2 weeks** | **Yes** | **HAMD-21** | **23.2±7.5** | **26.5±5.2** | **22.7±4.7** | **N.A.** | **6/10** | **2/12** | **1/11** | **N.A.** | **5/10** | **5/12** | **1/11** | **N.A.** | **9.8±7.1** | **12.3±7.7** | **19.0±7.7** | **N.A.** | **0/10** | **2/12** | **1/11** | **N.A.** |
| **2005** |  |  |  |  |  |  |  |  |  |  | **n=10** | **n=12** | **n=11** |  |  |  |  |  |  |  |  |  |  |  |  |  |  |  |  |  |  |  |  |  |  |  |  |
| **Huirong Zheng** | **China** | **No** | **34(22/12)** | **≥2/N.A.** | **26.9± 6.2** | **26.7± 4.3** | **N.A.** | **N.A.** | **No** | **No report** | **HFL sup-rTMS,3000 pulses per session** | **Sham-rTMS** | **N.A.** | **N.A.** | **4 weeks** | **Yes** | **HMAD-17** | **24.6±3** | **24.6±2.8** | **N.A.** | **N.A.** | **12/19** | **1/15** | **N.A.** | **N.A.** | **N.A.** | **N.A.** | **N.A.** | **N.A.** | **N.A.** | **N.A.** | **N.A.** | **N.A.** | **0/19** | **0/15** | **N.A.** | **N.A.** |
| **2010** |  |  |  |  |  |  |  |  |  |  | **n=19** | **n=15** |  |  |  |  |  |  |  |  |  |  |  |  |  |  |  |  |  |  |  |  |  |  |  |  |  |
| **Marie-Laure PaillereMartinot 2010** | **France** | **No** | **33(11/21)** | **≥2/N.A.** | **48.19± 7.77** | **46.57± 10.27** | **N.A.** | **N.A.** | **Yes** | **No report** | **HFL sub-rTMS，1600 pulses per session n=19** | **Sham-rTMS n=14** | **N.A.** | **N.A.** | **2 weeks** | **Yes** | **MADRS** | **32±7.78** | **34.57±6.07** | **N.A.** | **N.A.** | **10/19** | **3/14** | **N.A.** | **N.A.** | **N.A.** | **N.A.** | **N.A.** | **N.A.** | **N.A.** | **N.A.** | **N.A.** | **N.A.** | **1/19** | **0/14** | **N.A.** | **N.A.** |
| **Chris Baeken** | **Belgium** | **No** | **21(8/13)** | **≥3/N.A.** | **49.33±12.50** | | **N.A.** | **N.A.** | **No** | **No report** | **HFL sup-rTMS,1560 pulses per session** | **Sham-rTMS** | **N.A.** | **N.A.** | **1 week** | **No** | **HAMD17** | **24.75±7.1** | **26.45±8.7** | **N.A.** | **N.A.** | **2/10** | **1/11** | **N.A.** | **N.A.** | **0/10** | **0/11** | **N.A.** | **N.A.** | **19.63±8.3** | **22.36±10.0** | **N.A.** | **N.A.** | **2/10** | **0/11** | **N.A.** | **N.A.** |
| **2013** |  |  |  |  |  |  |  |  |  |  | **n=10** | **n=11** |  |  |  |  |  |  |  |  |  |  |  |  |  |  |  |  |  |  |  |  |  |  |  |  |  |
| **Nashaat N. Boutros** | **the United states** | **No** | **22(16/5)** | **≥2/N.A.** | **49.5±8** | **52±7** | **N.A.** | **N.A.** | **No** | **No** | **HFL sub-rTMS,800 stimuliy session** | **Sham-rTMS** | **N.A.** | **N.A.** | **2 weeks** | **Yes** | **HAMD-25** | **34.4±10.1** | **31.7±4.9** | **N.A.** | **N.A.** | **2/12** | **2/10** | **N.A.** | **N.A.** | **1/12** | **1/10** | **N.A.** | **N.A.** | **26.5±13.7** | **264±13.4** | **N.A.** | **N.A.** | **0/12** | **1/10** | **N.A.** | **N.A.** |
| **2002** |  |  |  |  |  |  |  |  |  |  | **n=12** | **n=10** |  |  |  |  |  |  |  |  |  |  |  |  |  |  |  |  |  |  |  |  |  |  |  |  |  |
| **Mauro Garcia-Toro** | **Spain** | **No** | **35(20/15)** | **≥2/N.A.** | **50.0±11.0** | **51.5±15.9** | **N.A.** | **N.A.** | **No** | **No report** | **HFL sub-rTMS** | **Sham-rTMS** | **N.A.** | **N.A.** | **2 weeks** | **Yes** | **HAMD-21** | **27.11±6.65** | **25.6±4.92** | **N.A.** | **N.A.** | **5/17** | **1/18** | **N.A.** | **N.A.** | **N.A.** | **N.A.** | **N.A.** | **N.A.** | **N.A.** | **N.A.** | **N.A.** | **N.A.** | **2/17** | **3/18** | **N.A.** | **N.A.** |
| **2001** |  |  |  |  |  |  |  |  |  |  | **n=17** | **n=18** |  |  |  |  |  |  |  |  |  |  |  |  |  |  |  |  |  |  |  |  |  |  |  |  |  |
| **Urs P. Mosimann** | **Switzerland** | **No** | **24(14/10)** | **≥2/N.A.** | **60.0 ±13.4** | **64.4 ±13.0** | **N.A.** | **N.A.** | **Yes** | **No report** | **HFL sub-rTMS 1600 pulses per session** | **Sham-rTMS** | **N.A.** | **N.A.** | **2 weeks** | **Yes** | **HAMD-21** | **28.5±4.6** | **24.5±7.2** | **N.A.** | **N.A.** | **1/15** | **0/9** | **N.A.** | **N.A.** | **N.A.** | **N.A.** | **N.A.** | **N.A.** | **23.3±7.2** | **20.4±6.6** | **N.A.** | **N.A.** | **0/15** | **0/9** | **N.A.** | **N.A.** |
| **2004** |  |  |  |  |  |  |  |  |  |  | **n=15** | **n=9** |  |  |  |  |  |  |  |  |  |  |  |  |  |  |  |  |  |  |  |  |  |  |  |  |  |
| **William J. Triggs** | **the United states** | **No** | **48(19/29)** | **≥2/N.A.** | **46.7± 15.3** | **48.5± 10.8** | **44.3± 16.4** | **N.A.** | **Yes** | **No** | **HFL sub-rTMS** | **HFR sub-rTMS** | **Sham-rTMS** | **N.A.** | **2 weeks** | **Yes** | **HAMD-24** | **27.2±4.8** | **28.2±6.0** | **27.5±3.0** | **N.A.** | **4/18** | **5/16** | **6/14** | **N.A.** | **N.A.** | **N.A.** | **N.A.** | **N.A.** | **19.8±9.1** | **13.7±7.6** | **17.7±10.4** | **N.A.** | **0/18** | **0/16** | **0/14** | **N.A.** |
| **2010** |  |  |  |  |  |  |  |  |  |  | **n=18** | **n=16** | **n=14** |  |  |  |  |  |  |  |  |  |  |  |  |  |  |  |  |  |  |  |  |  |  |  |  |
| **Paul E Holtzheimer** | **the United states** | **No** | **15(8/7)** | **≥2/N.A.** | **40.4± 8.5** | **45.4± 4.9** | **N.A.** | **N.A.** | **No** | **No** | **HFL sup-rTMS,1600 pulses per session** | **Sham-rTMS** | **N.A.** | **N.A.** | **2 weeks** | **No** | **HAMD-17** | **22.7±5.3** | **20.8±6.3** | **N.A.** | **N.A.** | **2/7** | **1/8** | **N.A.** | **N.A.** | **N.A.** | **N.A.** | **N.A.** | **N.A.** | **14.6±3.2** | **15.3±3.0** | **N.A.** | **N.A.** | **0/7** | **0/8** | **N.A.** | **N.A.** |
|  |  |  |  |  |  |  |  |  |  |  |  |  |  |  |  |  |  |  |  |  |  |  |  |  |  |  |  |  |  |  |  |  |  |  |  |  |  |
| **2004** |  |  |  |  |  |  |  |  |  |  | **n=7** | **n=8** |  |  |  |  |  |  |  |  |  |  |  |  |  |  |  |  |  |  |  |  |  |  |  |  |  |
| **Curtis D. Kauffmann** | **the United states** | **No** | **12(1/11)** | **≥2/N.A.** | **51.7±17.2** | | **N.A.** | **N.A.** | **No** | **No report** | **LFR-HFL sup-rTMS** | **Sham-rTMS** | **N.A.** | **N.A.** | **2 weeks** | **Yes** | **HAMD-21** | **21.86±2.31** | **18.20±2.20** | **N.A.** | **N.A.** | **4/7** | **2/5** | **N.A.** | **N.A.** | **N.A.** | **N.A.** | **N.A.** | **N.A.** | **21.86±2.31** | **11.29±3.17** | **N.A.** | **N.A.** | **0/7** | **0/5** | **N.A.** | **N.A.** |
| **2004** |  |  |  |  |  |  |  |  |  |  | **n=7** | **n=5** |  |  |  |  |  |  |  |  |  |  |  |  |  |  |  |  |  |  |  |  |  |  |  |  |  |
| **Shaw-Ji Chen** | **`China** | **No** | **20(9/12)** | **≥2/N.A.** | **44.1±4.4** | **47.3± 3.5** | **N.A.** | **N.A.** | **No** | **Yes** | **HFL sub-rTMS** | **Sham-rTMS** | **N.A.** | **N.A.** | **4 weeks** | **Yes** | **HAMD-17** | **23.5±1.9** | **24.9±1.9** | **N.A.** | **N.A.** | **7/10** | **8/11** | **N.A.** | **N.A.** | **N.A.** | **N.A.** | **N.A.** | **N.A.** | **9.6±1.5** | **12.3±1.4** | **N.A.** | **N.A.** | **0/10** | **0/11** | **N.A.** | **N.A.** |
|  |  |  |  |  |  |  |  |  |  |  |  |  |  |  |  |  |  |  |  |  |  |  |  |  |  |  |  |  |  |  |  |  |  |  |  |  |  |
| **2018** |  |  |  |  |  |  |  |  |  |  | **n=10** | **n=11** |  |  |  |  |  |  |  |  |  |  |  |  |  |  |  |  |  |  |  |  |  |  |  |  |  |
| **Bahadir Bakim** | **Turkey** | **No** | **35(4/31)** | **≥2/N.A.** | **43.09±8.18** | **38.75±9.96** | **44.41±10.22** | **N.A.** | **No** | **No** | **HFL sup-rTMS,800 pulses per session** | **HFL sub-rTMS,800 pulses per session** | **Sham-rTMS** | **N.A.** | **6 weeks** | **Yes** | **HAMD-17** | **24.09±2.77** | **23.08±3.63** | **25.58±3.82** | **N.A.** | **10/11** | **8/12** | **2/12** | **N.A.** | **6/11** | **3/12** | **2/12** | **N.A.** | **11.64±8.12** | **10.17±7.42** | **19.5±7.83** | **N.A.** | **0/11** | **0/12** | **0/12** | **N.A.** |
| **2012** |  |  |  |  |  |  |  |  |  |  | **n=11** | **n=12** | **n=12** |  |  |  |  |  |  |  |  |  |  |  |  |  |  |  |  |  |  |  |  |  |  |  |  |
| **David Rossini** | **Italy** | **No** | **54(16/38)** | **≥2/N.A.** | **57.4±8.7** | **54.0±11.2** | **56.3±12.6** | **N.A.** | **Yes** | **No** | **HFL sub-rTMS** | **HFL sub-rTMS** | **Sham-rTMS** | **N.A.** | **2 weeks** | **Yes** | **HAMD-21** | **28.8±3.1** | **28.6±2.7** | **28.7±2.1** | **N.A.** | **11/18** | **5/19** | **1/17** | **N.A.** | **9/18** | **5/19** | **0/17** | **N.A.** | **N.A.** | **N.A.** | **N.A.** | **N.A.** | **0/18** | **1/19** | **1/17** | **N.A.** |
| **2005** |  |  |  |  |  |  |  |  |  |  | **n=18** | **n=19** | **n=17** |  |  |  |  |  |  |  |  |  |  |  |  |  |  |  |  |  |  |  |  |  |  |  |  |
| **Christos Theleritis** | **Greece** | **No** | **98(50/48)** | **≥2/N.A.** | **39±12** | **38.8±9.5** | **N.A.** | **N.A.** | **No** | **No** | **HFL sub-rTMS,1600 pulses/session** | **Sham-rTMS** | **N.A.** | **N.A.** | **3 weeks** | **Yes** | **HAMD-17** | **30.2±4.0** | **29.9±3.4** | **N.A.** | **N.A.** | **29/54** | **1/44** | **N.A.** | **N.A.** | **12/54** | **0/44** | **N.A.** | **N.A.** | **14.4±4.3** | **26.3±4.6** | **N.A.** | **N.A.** | **4/54** | **5/44** | **N.A.** | **N.A.** |
|  |  |  |  |  |  |  |  |  |  |  |  |  |  |  |  |  |  |  |  |  |  |  |  |  |  |  |  |  |  |  |  |  |  |  |  |  |  |
| **2017** |  |  |  |  |  |  |  |  |  |  | **n=54** | **n=44** |  |  |  |  |  |  |  |  |  |  |  |  |  |  |  |  |  |  |  |  |  |  |  |  |  |
| **Jelena Krstić** | **Serbia** | **No** | **19(0/19)** | **≥2/N.A.** | **50.7 ±7.3** | **46.1±8.5** | **N.A.** | **N.A.** | **No** | **No** | **LFR-HFL sup-rTMS,300pulses per session** | **Sham-rTMS** | **N.A.** | **N.A.** | **2 weeks** | **Yes** | **HDRS-24** | **30.09± 3.53** | **28.00 ±2.74** | **N.A.** | **N.A.** | **4/11** | **0/8** | **N.A.** | **N.A.** | **N.A.** | **N.A.** | **N.A.** | **N.A.** | **17.5 ± 5.6** | **23.9 ± 3.8** | **N.A.** | **N.A.** | **0/11** | **0/8** | **N.A.** | **N.A.** |
|  |  |  |  |  |  |  |  |  |  |  |  |  |  |  |  |  |  |  |  |  |  |  |  |  |  |  |  |  |  |  |  |  |  |  |  |  |  |
| **2014** |  |  |  |  |  |  |  |  |  |  | **n=11** | **n=8** |  |  |  |  |  |  |  |  |  |  |  |  |  |  |  |  |  |  |  |  |  |  |  |  |  |
| **William M. McDonald, MD** | **Atlanta** | **No** | **219(28/40)** | **≥3/N.A.** | **47.5±11.3** | **46.9±12.3** | **47.2±11.8** | **N.A.** | **No report** | **No report** | **HFL sup-rTMS** | **Sham -rTMS** | **N.A.** | **N.A.** | **3-6 weeks** | **Yes** | **HAMD-24** | **26.1±5.3** | **26.5±4.6** | **N.A.** | **N.A.** | **N.A.** | **N.A.** | **N.A.** | **N.A.** | **14%（13/92）** | **5%（13/98）** | **N.A.** | **N.A.** | **24.6±5.9** | **24.5±6.0** | **N.A.** | **N.A.** | **N.A.** | **N.A.** | **N.A.** | **N.A.** |
|  |  |  |  |  |  |  |  |  |  |  |  |  |  |  |  |  |  |  |  |  |  |  |  |  |  |  |  |  |  |  |  |  |  |  |  |  |  |
| **2011** |  |  |  |  |  |  |  |  |  |  |  |  |  |  |  |  |  |  |  |  |  |  |  |  |  |  |  |  |  |  |  |  |  |  |  |  |  |
|  |  |  |  |  |  |  |  |  |  |  |  |  |  |  |  |  |  |  |  |  |  |  |  |  |  |  |  |  |  |  |  |  |  |  |  |  |  |
| **Keith Isenberg** | **the United states** | **No** | **28(12/16)** | **≥2/N.A.** | **55.57±9.71** | **43.36 ±9.72** | **N.A.** | **N.A.** | **Yes** | **No** | **LFR-HFL sup-rTMS** | **HFL sub-rTMS** | **N.A.** | **N.A.** | **4 weeks** | **No** | **HAMD-21** | **23.93±6.18** | **25.07 ±4.92** | **N.A.** | **N.A.** | **36%（5/14）** | **29%(4/14)** | **N.A.** | **N.A.** | **21%(3/14)** | **14%(2/14)** | **N.A.** | **N.A.** | **N.A.** | **N.A.** | **N.A.** | **N.A.** | **1/15** | **1/15** | **N.A.** | **N.A.** |
|  |  |  |  |  |  |  |  |  |  |  |  |  |  |  |  |  |  |  |  |  |  |  |  |  |  |  |  |  |  |  |  |  |  |  |  |  |  |
| **2005** |  |  |  |  |  |  |  |  |  |  | **n=14** | **n=14** |  |  |  |  |  |  |  |  |  |  |  |  |  |  |  |  |  |  |  |  |  |  |  |  |  |

**
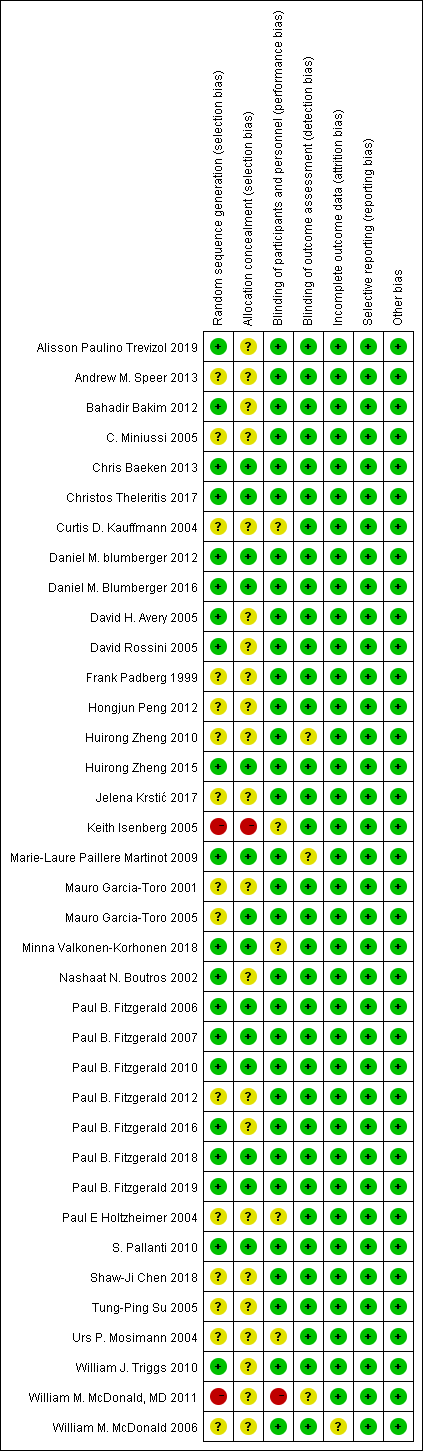
Appendix 3. Risk of bias assessment**


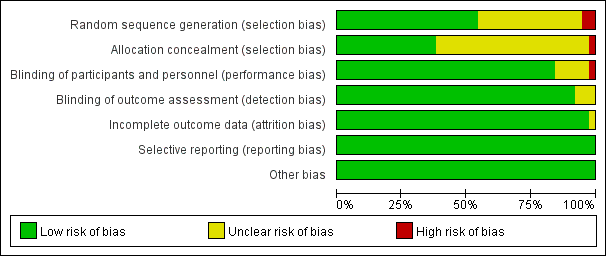

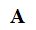


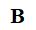


**Figure 1:** (A) Risk of bias for each included study; (B) Summary results of the bias risk assessment; Green, low risk of bias; Red, high risk of bias; Yellow, unclear risk of bias

**Table 3：Grading and support basis of bias risk**

|  | | | | | | | | | | | | | | | |
| --- | --- | --- | --- | --- | --- | --- | --- | --- | --- | --- | --- | --- | --- | --- | --- |
| Source | Random sequence generation | | Allocation concealment | | Blinding of participants and personnel | | Blinding of outcome assessment | | Incomplete outcome data | | Selective reporting | | Other bias | | over-all risk of bias |
|  | Grade | Support | Grade | Support | Grade | Support | Grade | Support | Grade | Support | Grade | Support | Grade | Support | Grade |
| Paul B. Fitzgerald | Low | Patients were randomized using a single computer-generated random number sequence | Low | A single computer-generated | Low | Patients and raters were blind to group. | Low | Raters were blind to group. | Low | 26 of 59 patients in group1，51 of 91 patients in group 2 ，15 of 57 patients in group 3，63 of 93 patients in group 4 discontinued intervention | Low | Primary outcome measure was antidepressant response (50% reduction of HRSD score) or remission (HRSD score of less than 8). | Low | None | Low |
| 2019 |  |  |  |  |  |  |  |  |  |  |  |  |  |  |  |
| Paul B. Fitzgerald | Low | Patients were randomized using a randomization code generated by a computer sequence. | Low | Stored in a sealed envelope | Low | Patients and raters were blind to group. | Low | Raters were blind to group. | Low | All eight patients completed the double-blind trial period. | Low | Primary outcome measure was antidepressant response (50% reduction of MADRS score) or remission (MADRS score of less than 10). | Low | None | Low |
| 2007 |  |  |  |  |  |  |  |  |  |  |  |  |  |  |  |
| Keith Isenberg | High | Based upon date of entry into the study | High | Based upon date of entry into the study | Unclear | No description | Low | All clinician ratings were blind to treatment status. | Low | Two patients (one in the active group, one in sham group) drop-out | Low | The primary outcome measure for the study was the hamd-21 | Low | None | High |
| 2005 |  |  |  |  |  |  |  |  |  |  |  |  |  |  |  |
| Alisson Paulino Trevizol | Low | A randomisation list was created | Unclear | No description | Low | Patients and raters were blind to group. | Low | evaluators were  blinded. | Low | Four patients dropped out (one in the bilateral group, two in theunilateral group and one in sham group. | Low | Primary outcome measure was antidepressant response rates(> 50%  reduction in hdrs scores) or remission (a score ≤ 10 on the 17- item hdrs). | Low | None | Low |
| 2019 |  |  |  |  |  |  |  |  |  |  |  |  |  |  |  |
| Paul B. Fitzgerald | Low | The patients were sequentially randomly assigned to groups with a single randomnumber sequence (no stratification) | Low | A series of sealed envelopes | Low | Patients and raters were blind to group. | Low | Raters were blind to group. | Low | Of the 50 patients randomly assigned to groups, three (all in the sham group) failed to complete the initial 2-week treatment period. | Low | The primary outcome measure was score on the MADRS. The patients were assessed with the MADRS, the HAM-D-17, the BDI, the BPRS, the CORE Rating of Psychomotor Disturbance , and the GAF Scale | Low | None | Low |
| 2006 |  |  |  |  |  |  |  |  |  |  |  |  |  |  |  |
| William M. McDonald | Unclear | Subjects were randomized to either sham TMS or a combination of Fast Left (10 Hz) rtms over the DLPFC or Slow Right in a 1:2:2 ratio, respectively | Unclear | No description | Low | The subject blind to the randomization | Low | A research assistant blind to the randomization completed all clinical measures | Unclear | No description | Low | The HDRS score was the primary measure of efficacy | Low | None | Low |
| 2006 |  |  |  |  |  |  |  |  |  |  |  |  |  |  |  |
| Paul B. Fitzgerald | Low | Patients were randomized using a single computer-generated random number sequence (no stratification). | Low | A single computer-generated | Low | Patients and raters were blind to group. | Low | Raters were blind to group. | Low | Fifty-nine patients dropped out (eleven in the group 1, twenty-five in group 2 and twenty-three in group 3）. | Low | Primary outcome measure was HAMD score. | Low | None | Low |
| 2010 |  |  |  |  |  |  |  |  |  |  |  |  |  |  |  |
| S. Pallanti | Low | Using computer database containing the randomization list. | Low | By an independent third party using a protected and concealed computer database | Low | The patients were blind to the allocated treatment. | Low | Ratings were performed by researchers blind to treatment | Low | None left the study | Low | The primary outcome measure was the ham-d | Low | None | Low |
| 2010 |  |  |  |  |  |  |  |  |  |  |  |  |  |  |  |
| William M. McDonald, MD | High | Phase 1 was a sham controlled randomized trial ; Phase 2 was an open label fast left rtms trial | Unclear | No description | High | Patients in Phase 2 received up  to 6 weeks of open label fast left rtms | Unclear | No description | Low | Thirty-one patients declined to continue into Phase 2 | Low | The primary outcome was scores on HAMD-17. In addition, all patients completed the MADRS and the BDI | Low | None | High |
| 2011 |  |  |  |  |  |  |  |  |  |  |  |  |  |  |  |
| DANIEL M. BLUMBERGER | Low | Individuals were randomized on a computer-generated list | Low | Information stored on a central to one of three treatment arms | Low | Subjects were blind to randomization group and instructed not to discuss their treatment with the clinical rater | Low | The clinical rater were blind to randomization group | Low | Data on the primary outcome was available for = 49 subjects (72.1%). Subjects who were lost to follow-up did not differ from retained subjects on any of the baseline clinical, cognitive or demographic variables. | Low | The primary outcome for the study was remission of depression (dichotomous outcome).remission was predefined as a final hrds score 10 . | Low | None | Low |
| 2012 |  |  |  |  |  |  |  |  |  |  |  |  |  |  |  |
| Andrew M. Speer | Unclear | No description | Unclear | No description | Low | Double-blind | Low | These raters were blind to both active versus sham treatment as well as to high versus low frequency of stimulation, | Low | All patients completed the study. | Low | The change in HAM-D ratings from baseline to after 3 weeks of each of the randomized treatments was assessed. | Low | None | Unclear |
| 2013 |  |  |  |  |  |  |  |  |  |  |  |  |  |  |  |
| Minna Valkonen-Korhonen | Low | Randomization was con- ducted by placing 21 labels with the text ‘TMS’ and 21 labels with the text ‘sham’ into identical envelopes | Low | Identical envelopes | Unclear | No description | Low | The rater was blind to the randomiza- tion setting of the rtms. | Low | Of the total sample, 2 of 18 patients in active rtms group and 1 of 19 patients in sham rtms group discontinued intervention | Low | Full remission(HAM-D score of less than 7) was the primary outcome measure. | Low | None | Low |
| 2018 |  |  |  |  |  |  |  |  |  |  |  |  |  |  |  |
| Paul B. Fitzgerald | Low | Randomization occurred using a single computer generated number sequence. | Low | A single computer generated number sequence. | Unclear | No description | Low | Raters were blind to treatment type. | Low | Of these 59 patients, 1 in the right-sided treatment group withdrew after treatments | Low | The primary outcome measure was score on the MADRS. | Low | None | Low |
| 2018 |  |  |  |  |  |  |  |  |  |  |  |  |  |  |  |
| C. Miniussi | Unlcear | Patients were randomly assigned to the two groups of TMS- treatment: group 17R for high-frequency (17 Hz) TMS and group 1R for low-frequency (1 Hz) TMS. | Unclear | No description | Unclear | No description | Low | An expert psychiatrist blind to the treatment performed the ratings. | Low | No one drop-out from the study | Low | The primary analysis was based on HRSD scores. | Low | None | Unclear |
| 2005 |  |  |  |  |  |  |  |  |  |  |  |  |  |  |  |
| Frank Padberg | Unclear | Patients were randomly assigned to three treatment groups. | Unclear | No description | Low | Double-blind,patients were not familiar with the differences between sham and verum rtms regarding acoustic and tactile artifacts | Low | Raters were experienced psychiatrists and blind to stimulation conditions. | Low | All subject comlpeted the study | Low | Severity of depression was rated with hamd-21 and madrs | Low | None | Unclear |
| 1999 |  |  |  |  |  |  |  |  |  |  |  |  |  |  |  |
| Daniel M. Blumberger | Low | Participants were randomized using a computer-generated list with a permuted, random block design, | Low | No description | Low | Clinical evaluators and participants were all blinded to the treatment condition. | Low | Clinical evaluators and participants were all blinded to the treatment condition. | Low | 16 of 121 participants  did not complete an end point assessment for the pri- mary outcome: 4 of 40 in the bilateral group, 7 of 40 in the unilateral group and 5 of 41 in the sham  group | Low | The primary outcome measure was the remission rate, with remission defined as a score of 7 or less on the hamd-17 at the week 3 or week 6 assessment. | Low | None | Low |
| 2016 |  |  |  |  |  |  |  |  |  |  |  |  |  |  |  |
| Mauro Garcia-Toro | Unclear | No descdription | Low | Using closed envelopes | Low | Patients were blind to the treatment condition. | Low | Raters were blind to the treatment condition. | Low | All subjects completed the entire study | Low | For assessment of antidepressant effects, we used the HAMD-21 and thegci. | Low | None | Low |
| 2005 |  |  |  |  |  |  |  |  |  |  |  |  |  |  |  |
| David H. Avery | Low | Randomization was performed with a computer program | Unclear | No description | Low | Subjects were blind to treatment allocation throughout the entire treatment protocol; | Low | The raters, who were never the treaters, were blind to treatment allocation and did not ask the subjects about side effects | Low | No subject dropped out because of pain or discomfort of the TMS treatment | Low | Response was defined as a 50% decrease in HDRS score from baseline to visit 16 that persisted at visit 17. Remission was defined as a HDRS 8 at visit 16 that persisted at visit 17. | Low | None | Low |
| 2005 |  |  |  |  |  |  |  |  |  |  |  |  |  |  |  |
| Paul B. Fitzgerald | Unclear | Patients were sequentially randomised with no stratification | Unclear | No description | Low | The patients were blind to treatment | Low | The raters were blind to treatment | Low | Six patients (three in group1, three in group 3, none in group2) withdrew during the initial three week period of double blind treatment | Low | The primary outcome was scores on hamd-17. In addition, all patients completed the madrs and the bdi | Low | None | Unclear |
| 2012 |  |  |  |  |  |  |  |  |  |  |  |  |  |  |  |
| Paul B. Fitzgerald | Low | Randomisation occurred through the use of a single random number sequence | Unclear | No description | Low | Patients were blind to group. | Low | Raters were blind to group. | Low | Of the 46 randomisedsubjects,six (4 active, 2 sham) with drew during the initial four-week period of double blind treatment | Low | The primary outcome variable was scores on HAMD-17 from baseline to week4. | Low | None | Low |
| 2016 |  |  |  |  |  |  |  |  |  |  |  |  |  |  |  |
| Huirong Zheng | Low | A two-group randomized blinded (patients and rater) trial by sequentially numbered containers. | Low | By sequentially numbered containers | Low | Blinded (patients and rater) | Low | Blinded (patients and rater) | Low | No one drop-out from the study | Low | Clinical symptoms were assessed by hamd-17, bdi and psqi. Response was defined as a hamd reduction of 50% from baseline scores. | Low | None | Low |
| 2015 |  |  |  |  |  |  |  |  |  |  |  |  |  |  |  |
| Tung-Ping Su | Unclear | Noc description | Unclear | No description | Low | Double-blind | Low | Severity of depression was assessed by a psychiatrist, blinded to treatment arm | Low | Severity of depression was assessed using hamd-21, ham-a, cgi-s scale.response was definied as a more than 50% reduction of hamd-21 scores... | Low | Three of thirty-three patients dropped-out, two in the active group, one in the sham group. | Low | None | Unclear |
| 2005 |  |  |  |  |  |  |  |  |  |  |  |  |  |  |  |
| Huirong Zheng | Unclear | No description | Unclear | No description | Low | Sham stimulation occurred in exactly the same manner as active rtms | Unclear | Double-blind, no description | Low | All subjects completed the study | Low | Clinical symptoms were assessed by the hamd-17 and the bdi | Low | None | Unclear |
| 2010 |  |  |  |  |  |  |  |  |  |  |  |  |  |  |  |
| Marie-Laure PaillereMartinot 2009 | Low | Randomization was stratified on the | Low | Allocation concealment was performed using closed envelopes | Low | The patients were blind to the treatment modality and had never previously been treated with TMS | Unclear | Double-blind, no details | Low | One subject in active group droped-out from the study | Low | Clinical evaluations were performed using the MADRS, HAMD21, and thecgi-S. | Low | None | Low |
|  |  | Stimulation site and two allocation lists were generated by the Biostatistics Department |  |  |  |  |  |  |  |  |  |  |  |  |  |
| Chris Baeken | Low | Flipping a coin | Low | Flipping a coin | Low | Patients were kept unaware of the type of stimulation;they wore earplugs and were blindfolded. | Low | Depression severity was assessed by a certified psychiatrist, unrelated to the actual hf-rtms treatment of the patient | Low | Because of clinical non-response, one female patient,(real hf-rtms) refused to continue treatment. | Low | Depression severity was assessed with the hamd-17. Defined clinical response as a 50% reduction of the baseline hdrs score | Low | None | Low |
| 2013 |  |  |  |  |  |  |  |  |  |  |  |  |  |  |  |
| Nashaat N. Boutros | Low | Randomization was done using a computer-generated sequence | Unclear | No description | Low | Double-blind, the unblinded psychiatrist who administered the TMS had minimal interaction with patients. | Low | The same fully trained research assistant, who was kept blind to the treatment condition, administered all the hamds. | Low | One of the patients randomized to sham rtmsdropped out following the first session | Low | Weekly HAMD interviews were mainly performed in person. | Low | None | Low |
| 2002 |  |  |  |  |  |  |  |  |  |  |  |  |  |  |  |
| Mauro Garcia-Toro | Unclear | No description | Unclear | No description | Low | Patients were unaware to which procedure was used. | Low | Three clinicians who assessed efficacy were unaware to which procedure was used. | Low | Five patients did not complete the 4 weeks follow-up in the first phase. Two patients in the sham sham and three patient in the active group | Low | The efficacy measures used were the HAMD-21, the HARS, the CGI and the BDI. | Low | None | Unclear |
| 2001 |  |  |  |  |  |  |  |  |  |  |  |  |  |  |  |
| Urs P. Mosimann | Unclear | No description | Unclear | No description | Unclear | No description | Low | Outcome ratings were assessed on a different floor of the building by a blinded rater | Low | All patients completed the study | Low | Severity of depression was assessed using four different depression scales. The HAMD-21 was the primary outcome measure. | Low | None | Unclear |
| 2004 |  |  |  |  |  |  |  |  |  |  |  |  |  |  |  |
| William J. Triggs | Low | Subjects were randomized 1:1:1 to receive left frontal rtms, right frontal rtms, or sham rtms. | Unclear | No description | Low | Double-blind, sham group is in an effort to simulate the level of discomfort observed during real rtms | Low | The mood assessments were administered by trained psychiatry research nurses unaware of the patient's treatment group (real or sham rtms). | Low | All subjects completed the post-treatment evaluation | Low | We rated mood using the HAMD0-24 and the long form of the BDI. We used the STAI as a secondary measure.. | Low | None | Low |
| 2010 |  |  |  |  |  |  |  |  |  |  |  |  |  |  |  |
| Paul E Holtzheimer | Unclear | Subjects were randomly assigned to receive either active or sham rtms,no more details | Unclear | No description | Unclear | No description | Low | Blind raters administered the HDRS | Low | All 15 subjects completed all ten treatment sessions in the blinded portion of the study. | Low | The HDRS and the BDI were completed, and response to rtms was defined as a >50% decrease in HDRS at week 3. | Low | None | Unclear |
| 2004 |  |  |  |  |  |  |  |  |  |  |  |  |  |  |  |
| Curtis D. Kauffmann | Unclear | All subjects were randomly assigned to receive rtms or sham rtms in a double-blind design, no more details | Unclear | No description | Unclear | The method for the sham treatment group has been found to give the same sensation in the scalp as the real treatment | Low | The rater was a resident psychiatrist who was involved in the diagnostic evaluation but was unaware of the nature of treatment | Low | All of the 12 patients completed the study | Low | Clinical ratings were assessed at baseline , after five sessions (1 week), and at the last session using the HAMD scale | Low | None | Unclear |
| 2004 |  |  |  |  |  |  |  |  |  |  |  |  |  |  |  |
| Shaw-Ji Chen | Unclear | Study participants were randomized into two groups, no more details | Unclear | No description | Low | Double blind, Sham stimulation occurred in exactly the same manner as active rtms, | Low | The raters who evaluated the patients did not know whether a participant had been assigned to the rtms or sham group | Low | One patient in the sham group withdrew from the study because of unspecified somatic complaints | Low | Two scales were used to evaluate the efficacy of rtms in medication-resistant depression: the BDI-II and the 17-item HAM-D. | Low | None | Unclear |
| 2013 |  |  |  |  |  |  |  |  |  |  |  |  |  |  |  |
| Bahadir Bakim | Low | Using a random allocation software | Unclear | No description | Low | Doublind, sham stimulation gave patients a similar sound effect | Low | Both raters who were experienced psychiatrists and the participants were all blind to the stimulation parameters | Low | All subjects completed the study | Low | Patients were assessed using the hamd-17 and madrs .response was defined as a 50% or above decrease... | Low | None | Low |
| 2012 |  |  |  |  |  |  |  |  |  |  |  |  |  |  |  |
| David Rossini | Low | According to a computer-generated random list | Unclear | No description | Low | Double-blind the, all patients declared themselves to be unaware of the differences between sham and active stimulation | Low | The assessment was performed by two trained psychiatrists who were unaware of the stimulation parameters. | Low | Of the 54 enrolled patients, 52 completed the entire protocol. Two patients dropped out... | Low | Assessment was performed using the hamd-21 and the cgi-s and cgi-i | Low | None | Low |
| 2005 |  |  |  |  |  |  |  |  |  |  |  |  |  |  |  |
| Christos Theleritis | Low | Using the randomization list. | Low | Using a passwordprotected computer database containing the randomization list | Low | Patients and raters were blind to allocated treatment | Low | Patients and raters were blind to allocated treatment | Low | Eighty-nine subjects completed the 5-week trial and 9 (9%) discontinued (2 from a1 group, 2 from a2 group, 2 from s1 group, and 3 froms2 group | Low | Outcome measures were the hamd-17 and the cgi-s. | Low | None | Low |
| 2017 |  |  |  |  |  |  |  |  |  |  |  |  |  |  |  |
| Jelena Krstić | Unclear | Conducting a blind randomized study of active and sham right prefrontal rtms in patients with treatment-resistant MD. | Unclear | No description | Low | The patients and the rater were blind to the treatment | Low | The patients and the rater were blind to the treatment | Low | All subjects completed the 2 weeks study | Low | Outcome measures were the HAMD-24 and the CGI-S. | Low | None | Unclear |
| 2017 |  |  |  |  |  |  |  |  |  |  |  |  |  |  |  |
| Hongjun Peng | Unclear | 17 patients were treated with active rtms, while 13 patients were trea- ted with sham rtms, selected at random. . | Unclear | No description | Low | Blinded (patients and rater) | Low | The researcher was unaware of the rtms condition (active or sham) until completing the second DTI scan following 4 weeks of rtms treatment. | Low | No one drop-out from the study | Low | A series of two-way repeated measures anovas was conducted to compare changes in BDI, HAMD scores and FA values between pre- and post- treatment in active and sham stimulation groups, | Low | None | Unclear |
| 2012 |  |  |  |  |  |  |  |  |  |  |  |  |  |  |  |

**Appendix 4. Results from pairwise meta-analyses**


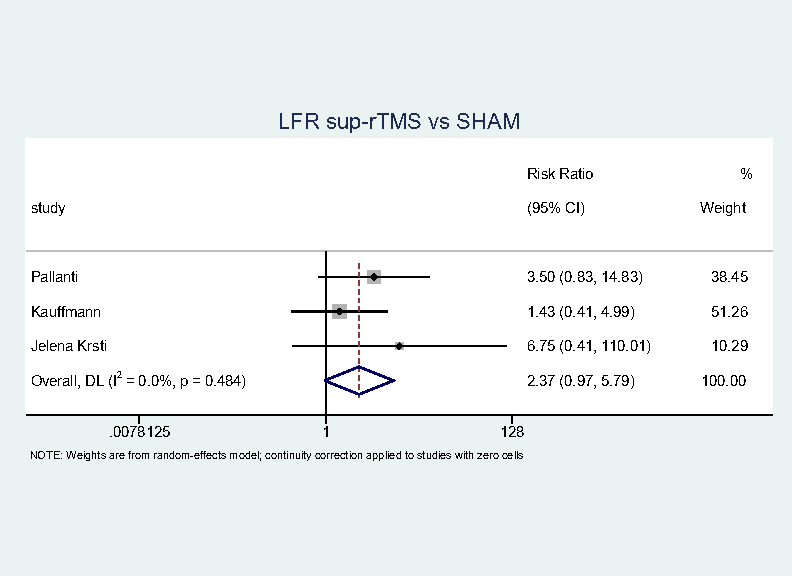

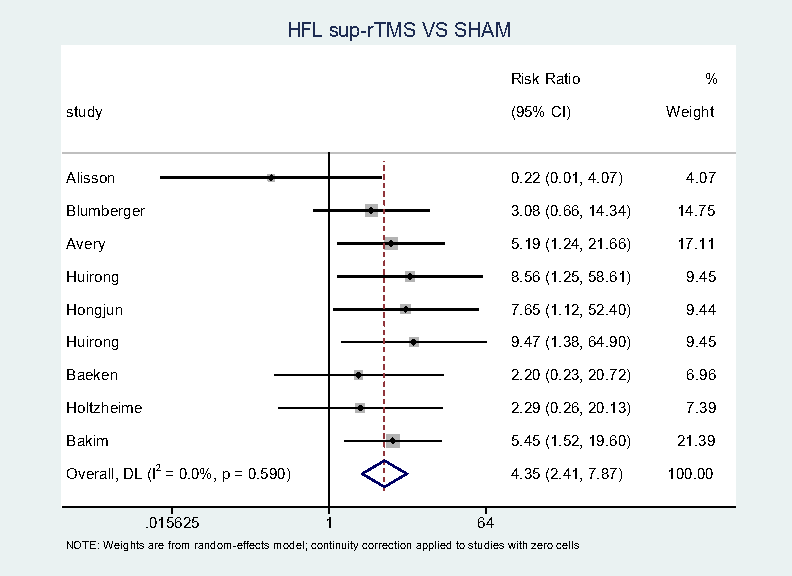


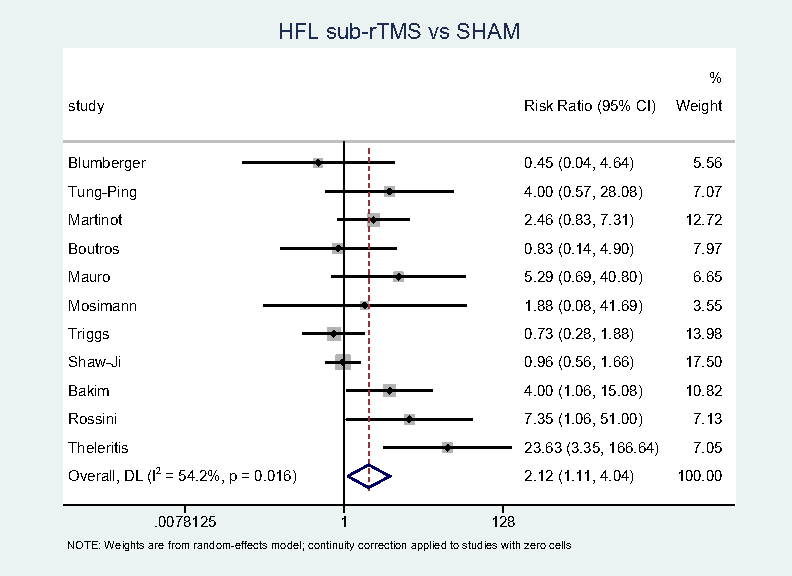

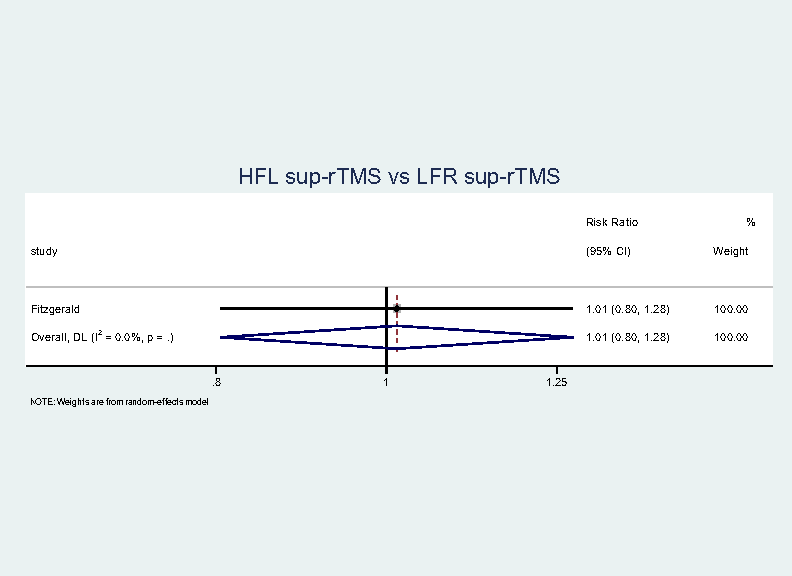


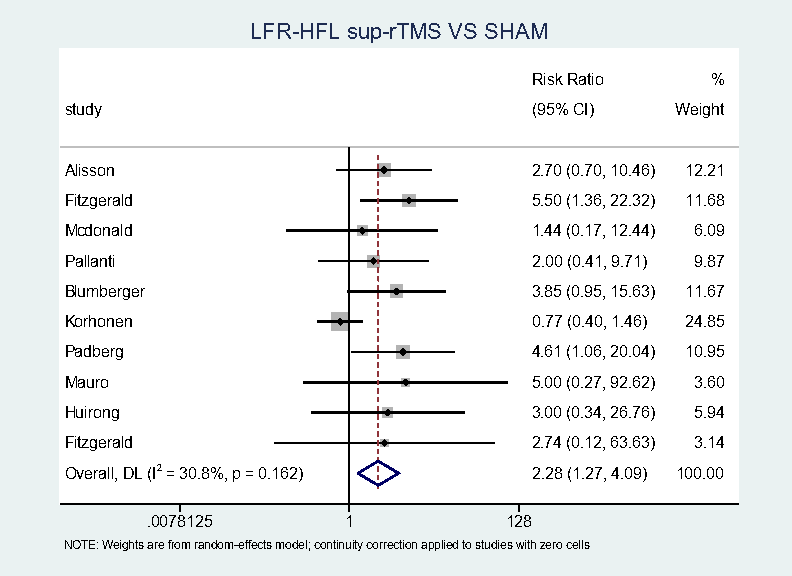

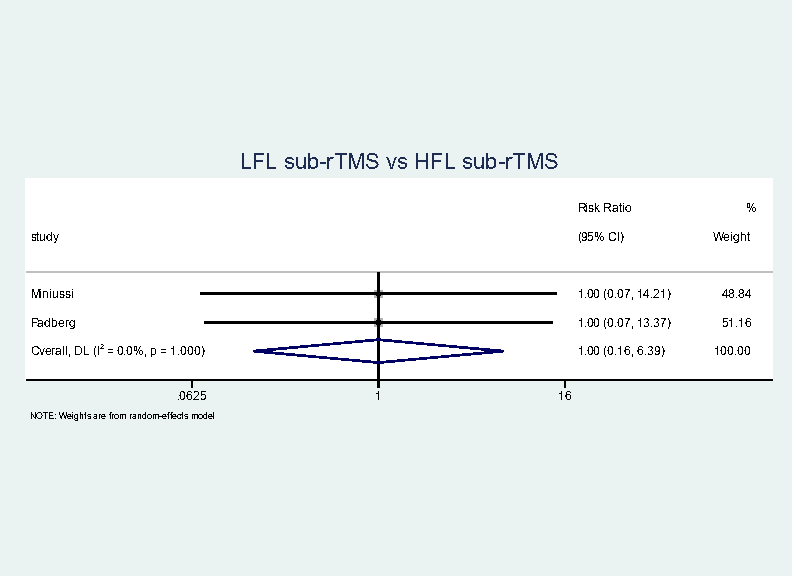


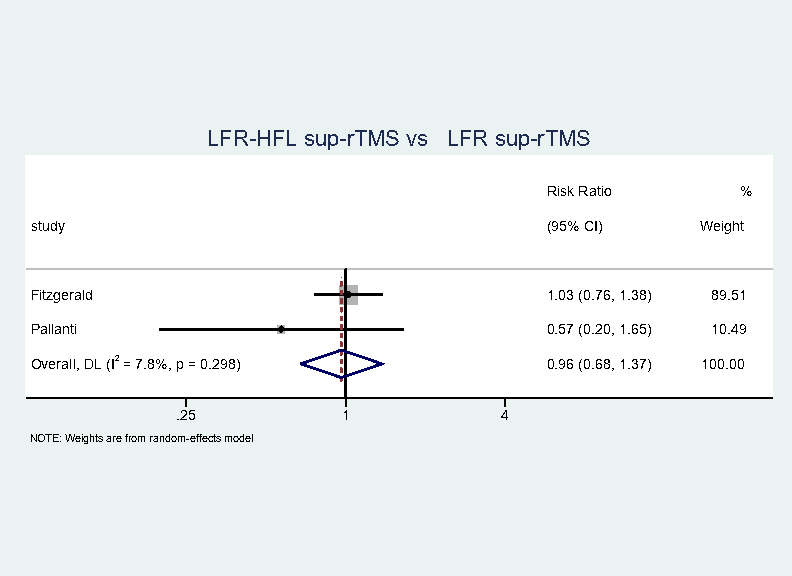

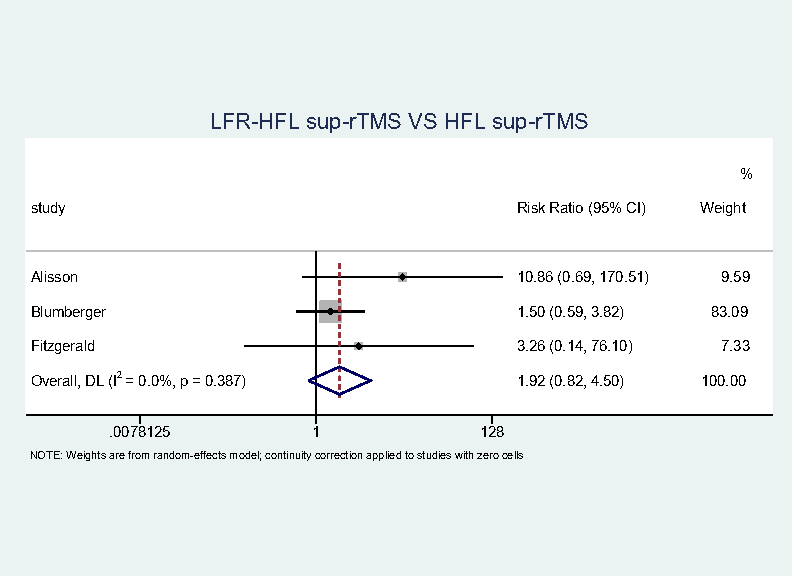


**Figure 2：**Forest plot of the response rate: LFRsup-rTMS vs SHAM; HFLsup-rTMS vs SHAM; HFLsup-rTMS vs LFRsup-rTMS; HFLsub-rTMS vs SHAM; LFLsub-rTMS vs HFLsub-rTMS ; LFR-HFLsup-rTMS vs SHAM; LFR-HFL-rTMS vs LFRsup-rTMS; LFR-HFLsup-rTMS vs HFLsup-rTMS. The right-hand side of value 1 suggests that the former Intervention was more efficacious.

**Table 4: Summary of pairwise meta-analyses for response**

|  |  | Respond | | | |  |  | Respond | | | |
| --- | --- | --- | --- | --- | --- | --- | --- | --- | --- | --- | --- |
|  | Study | RR | 95% CI | | I^2^ |  | Study | RR | 95% CI | | I^2^ |
| HFL sup-rTMS vs SHAM | Alisson Paulino Trevizol 2019 | 0.22 | 0.01 | 4.07 | N.A. | LFR-HFL sup-rTMS vs LFR sup-rTMS | Paul B. Fitzgerald 2010 | 0.98 | 0.73 | 1.31 | N.A. |
|  | Daniel M. Blumberger 2016 | 3.08 | 0.66 | 14.34 | N.A. |  | S. Pallanti 2010 | 1.57 | 0.61 | 5.05 | N.A. |
|  | David H. Avery 2005 | 5.19 | 1.24 | 21.66 | N.A. |  | Pooled Results | 1.04 | 0.72 | 1.52 | 10.9% |
|  | Huirong Zheng 2010 | 8.56 | 1.25 | 58.61 | N.A. | LFR-HFL sup-rTMS vs HFL sup-rTMS | Alisson Paulino Trevizol 2019 | 10.86 | 0.69 | 170.51 | N.A. |
|  | Hongjun Peng 2012 | 7.65 | 1.12 | 52.4 | N.A. |  | Daniel M. Blumberger 2016 | 1.50 | 0.59 | 3.82 | N.A. |
|  | Huirong Zheng 2015 | 9.47 | 1.38 | 64.9 | N.A. |  | Paul B. Fitzgerald 2012 | 3.26 | 0.14 | 76.1 | N.A. |
|  | Chris Baeken 2013 | 2.20 | 0.23 | 20.72 | N.A. |  | Pooled Results | 2.11 | 0.75 | 5.96 | 9.8% |
|  | Paul E Holtzheimer2014 | 2.29 | 0.26 | 20.13 | N.A. | LFL sub-rTMS vs HFL sub-rTMS | C. Miniussi 2005 | 1.00 | -2.99 | 4.99 | N.A. |
|  | Bahadir Bakim 2012 | 5.45 | 1.52 | 19.6 | N.A. |  | Frank Padberg 1999 | 1.00 | -3.03 | 5.03 | N.A. |
|  | Pooled Results | 4.35 | 2.41 | 7.87 | 0.00% |  | Pooled Results | 1.00 | -1.84 | 3.84 | 0.00% |
| HFL sup-rTMS vs LFR sup-rTMS | Paul B. Fitzgerald 2019 | 1.01 | -0.94 | 2.96 | N.A. | LFR-HFL sup-rTMS vs SHAM | Alisson Paulino Trevizol 2019 | 2.70 | 0.70 | 10.46 | N.A. |
|  | Paul B. Fitzgerald 2018 | 0.86 | -3.09 | 4.82 | N.A. |  | Paul B. Fitzgerald 2012 | 5.50 | 1.36 | 22.32 | N.A. |
|  | Pooled Results | 0.98 | -0.76 | 2.73 | 0% |  | William M. McDonald 2006 | 1.44 | 0.17 | 12.44 | N.A. |
| HFL sub-rTMS vs SHAM | Daniel M. Blumberger 2012 | 0.45 | 0.04 | 4.64 | N.A. |  | S. Pallanti 2010 | 2.00 | 0.41 | 9.71 | N.A. |
|  | Tung-Ping Su 2005 | 4.00 | 0.57 | 28.08 | N.A. |  | Daniel M. Blumberger 2012 | 3.85 | 0.95 | 15.63 | N.A. |
|  | Marie-Laure PaillereMartinot 2009 | 2.46 | 0.83 | 7.31 | N.A. |  | Minna Valkonen-Korhonen 2018 | 0.77 | 0.40 | 1.46 | N.A. |
|  | Nashaat N. Boutros 2002 | 0.83 | 0.14 | 4.9 | N.A. |  | Daniel M. Blumberger 2016 | 4.61 | 1.06 | 20.04 | N.A. |
|  | Mauro Garcia-Toro 2001 | 5.29 | 0.69 | 40.8 | N.A. |  | Mauro Garcia-Toro 2005 | 5.00 | 0.27 | 92.62 | N.A. |
|  | Urs P. Mosimann 2004 | 1.88 | 0.08 | 41.69 | N.A. |  | Paul B. Fitzgerald 2016 | 3.00 | 0.34 | 26.76 | N.A. |
|  | William J. Triggs 2010 | 0.73 | 0.28 | 1.88 | N.A. |  | Paul B. Fitzgerald 2012 | 2.74 | 0.12 | 63.63 | N.A. |
|  | Shaw-Ji Chen 2018 | 0.96 | 0.56 | 1.66 | N.A. |  | Pooled Results | 2.36 | 1.26 | 4.40 | 38.2% |
|  | Bahadir Bakim 2012 | 4.00 | 1.06 | 15.08 | N.A. | LFR sup-rTMS vs SHAM | Pallanti 2010 | 3.50 | 0.83 | 14.83 | N.A. |
|  | David Rossini 2005 | 7.35 | 1.06 | 51 | N.A. |  | Kauffmann 2004 | 1.43 | 0.41 | 4.99 | N.A. |
|  | Christos Theleritis 2017 | 23.63 | 3.35 | 166.64 | N.A. |  | Jelena Krsti2017 | 6.75 | 0.41 | 110.01 | N.A. |
|  | Pooled Results | 2.24 | 1.03 | 4.87 | 68.8% |  | Pooled RR | 2.37 | 0.97 | 5.79 | 0.00% |

**Table 5: Summary of pairwise meta-analyses for the discontinuation rate**

|  |  | Discontinues | | | |  |  | Discontinues | | | |
| --- | --- | --- | --- | --- | --- | --- | --- | --- | --- | --- | --- |
|  | study | RR | 95%CI | | I2 |  | study | RR | 95%CI | | I2 |
| LFR sup-rTMS vs SHAM | S. Pallanti 2010 | 1.00 | -2.96 | 4.96 | N.A. | HFL sub-rTMS vs SHAM | Daniel M. Blumberger 2012 | 1.74 | 0.09 | 3.40 | N.A. |
|  | Curtis D. Kauffmann 2004 | 0.75 | -3.28 | 4.78 | N.A. |  | Frank Padberg 1999 | 1.00 | -3.03 | 5.03 | N.A. |
|  | Jelena Krstić 2017 | 0.78 | -3.21 | 4.78 | N.A. |  | Tung-Ping Su 2005 | 0.87 | -1.33 | 3.07 | N.A. |
|  | Pooled RR | 0.85 | -1.46 | 3.15 | 0.00% |  | Marie-Laure PaillereMartinot 2009 | 2.25 | -1.00 | 5.5 | N.A. |
| LFR sup-rTMSvs HFL sup-rTMS | Paul B. Fitzgerald 2019 | 1.01 | 0.81 | 1.26 | N.A. |  | Nashaat N. Boutros 2002 | 0.28 | -3.06 | 3.62 | N.A. |
|  | Paul B. Fitzgerald 2018 | 3.47 | 0.15 | 80.35 | N.A. |  | Mauro Garcia-Toro 2001 | 0.75 | -1.18 | 2.69 | N.A. |
|  | Pooled RR | 1.02 | 0.82 | 1.27 | 0.00% |  | Urs P. Mosimann 2004 | 0.63 | -3.35 | 4.6 | N.A. |
| LFR sup-rTMS vs LFR-HFL sup-rTMS | Paul B. Fitzgerald 2010 | 2.04 | 0.90 | 3.19 | N.A. |  | William J. Triggs 2010 | 0.88 | -3.08 | 4.85 | N.A. |
|  | S. Pallanti 2010 | 1.00 | -2.96 | 4.96 | N.A. |  | Shaw-Ji Chen 2018 | 1.09 | -2.90 | 5.08 | N.A. |
|  | Paul B. Fitzgerald 2018 | 3.32 | 0.07 | 6.56 | N.A. |  | Bahadir Bakim 2012 | 1.00 | -2.98 | 4.98 | N.A. |
|  | Pooled RR | 2.10 | 1.06 | 3.14 | 0.00% |  | David Rossini 2005 | 0.47 | -1.89 | 2.84 | N.A. |
| HFL sup-rTMS vs SHAM | Alisson Paulino Trevizol 2019 | 1.81 | -0.43 | 4.04 | N.A. |  | Christos Theleritis 2017 | 0.67 | -0.80 | 2.14 | N.A. |
|  | Daniel M. Blumberger 2016 | 1.40 | 0.02 | 2.78 | N.A. |  | Pooled RR | 0.98 | 0.27 | 1.69 | N.A. |
|  | David H. Avery 2005 | 0.94 | -0.72 | 2.60 | N.A. | HFL sub-rTMSvs LFL sub-rTMS | C. Miniussi 2005 | 1.00 | -2.99 | 4.99 | N.A. |
|  | Paul B. Fitzgerald 2012 | 0.12 | -3.00 | 3.24 | N.A. |  | Frank Padberg 1999 | 1.00 | -3.03 | 5.03 | N.A. |
|  | Huirong Zheng 2015 | 0.79 | -3.17 | 4.75 | N.A. |  | Pooled RR | 1.00 | -1.84 | 3.84 | 0.00% |
|  | Hongjun Peng 2012 | 0.78 | -3.19 | 4.74 | N.A. | LFR-HFL sup-rTMS vs SHAM | Alisson Paulino Trevizol 2019 | 0.62 | -1.79 | 3.03 | N.A. |
|  | Huirong Zheng 2010 | 0.80 | -3.16 | 4.76 | N.A. |  | Paul B. Fitzgerald 2006 | 0.14 | -2.94 | 3.23 | N.A. |
|  | Chris Baeken 2013 | 5.45 | 2.32 | 8.59 | N.A. |  | S. Pallanti 2010 | 1.00 | -2.96 | 4.96 | N.A. |
|  | Paul E Holtzheimer 2004 | 1.13 | -2.89 | 5.14 | N.A. |  | Daniel M. Blumberger 2012 | 0.64 | -1.12 | 2.39 | N.A. |
|  | Bahadir Bakim 2012 | 1.08 | -2.9 | 5.06 | N.A. |  | Minna Valkonen-Korhonen 2018 | 1.75 | -0.40 | 3.91 | N.A. |
|  | Andrew M. Speer 2013 | 1.00 | -3.01 | 5.01 | N.A. |  | Daniel M. Blumberger 2016 | 0.84 | -0.66 | 2.33 | N.A. |
|  | Pooled RR | 1.41 | 0.64 | 2.18 | 0.00% |  | Mauro Garcia-Toro 2015 | 1.00 | -2.99 | 4.99) | N.A. |
| LFR-HFL sup-rTMS vs HFL sup-rTMS | Alisson Paulino Trevizol 2019 | 0.28 | -2.34 | 2.89 | N.A. |  | Paul B. Fitzgerald 2012 | 0.91 | -0.86 | 2.69 | N.A. |
|  | Daniel M. Blumberger 2016 | 0.57 | -0.99 | 2.13 | N.A. |  | Paul B. Fitzgerald 2016 | 1.80 | 0.06 | 3.54 | N.A. |
|  | Pooled RR | 0.49 | -0.85 | 1.83 | 0.00% |  | Pooled RR | 1.03 | 0.33 | 1.73 | 0.00% |

**Table 6: Summary of pairwise meta-analyses for the remission rate**

|  |  | Remission | | | |  |  | Remission | | | |
| --- | --- | --- | --- | --- | --- | --- | --- | --- | --- | --- | --- |
|  | study | RR | 95% CI | | I2 |  | study | RR | 95% CI | | I2 |
| HFL sup-rTMSva SHAM | Andrew M. Speer 2013 | 0.91 | 0.06 | 13.59 | N.A. | LFR-HFL sup-rTMS vs SHAM | Alisson Paulino Trevizol 2019 | 7.62 | 1.04 | 55.51 | N.A. |
|  | Daniel M. Blumberge 2016 | 3.08 | 0.33 | 28.34 | N.A. |  | Daniel M. Blumberger 2016 | 2.67 | 0.76 | 9.33 | N.A. |
|  | David H. Avery 2005 | 6.60 | 0.86 | 50.79 | N.A. |  | Pooled RR | 3.59 | 1.25 | 10.37 | 0.00% |
|  | Bahadir Bakim 2012 | 3.27 | 0.83 | 12.95 | N.A. | HFL sub-rTMS vs SHAM | Nashaat N. Boutros 2002 | 0.83 | 0.06 | 11.70 | N.A. |
|  | Pooled RR | 3.22 | 1.24 | 8.32 | 0.00% |  | Bahadir Bakim 2005 | 1.50 | 0.30 | 7.43 | N.A. |
| LFR-HFL sup-rTMS vs SHAM | Paul B. Fitzgerald 2006 | 19.00 | 1.17 | 309.77 | N.A. |  | David Rossini 2012 | 13.74 | 0.87 | 217.66 | N.A. |
|  | S. Pallanti 2010 | 2.00 | 0.20 | 20.33 | N.A. |  | Tung-Ping Su 2005 | 5.00 | 0.73 | 34.24 | N.A. |
|  | Daniel M. Blumberger 2012 | 6.92 | -0.95 | 50.24 | N.A. |  | Christos Theleritis 2017 | 20.45 | 1.24 | 336.07 | N.A. |
|  | Minna Valkonen-Korhonen 2018 | 0.70 | 0.24 | 2.09 | N.A. |  | Daniel M. Blumberger 2012 | 7.62 | 1.04 | 55.51 | N.A. |
|  | Paul B. Fitzgerald 2016 | 0.20 | -0.01 | 3.95 | N.A. |  | Pooled RR | 4.02 | 1.62 | 9.94 | 6.10% |
|  | Alisson Paulino Trevizol 2019 | 10.52 | 0.66 | 167.45 | N.A. | LFR sup-rTMS vs LFR-HFL sup-rTMS | Paul B. Fitzgerald 2010 | 0.88 | 0.55 | 1.41 | N.A. |
|  | Pooled RR | 2.42 | 0.65 | 8.97 | 54.50% |  | S. Pallanti 2010 | 3.00 | 0.69 | 13.12 | N.A. |
|  |  |  |  |  |  |  | Pooled RR | 1.32 | 0.43 | 4.09 | 58.50% |

**Table 7: Summary of pair-wise meta-analyses for the endpoint depression score**

|  |  | Endpoint Scores | | | |  |  | Endpoint Scores | | | |
| --- | --- | --- | --- | --- | --- | --- | --- | --- | --- | --- | --- |
|  | study | SMD | 95%CI | | I2 |  | study | SMD | 95%CI | | I2 |
| LFR sup-rTMS vs SHAM | Curtis D. Kauffmann 2004 | 3.93 | 1.86 | 6 | N.A. | HFL sub-rTMS vs SHAM | Frank Padberg 1999 | -0.12 | -1.25 | 1.01 | N.A. |
|  | Jelena Krstić 2017 | -1.3 | -2.31 | -0.29 | N.A. |  | Tung-Ping Su 2005 | -1.06 | -1.84 | -0.29 | N.A. |
|  | Pooled RR | 1.24 | -3.88 | 6.36 | 94.90% |  | William J. Triggs 2010 | -0.44 | -1.17 | 0.28 | N.A. |
| HFL sup-rTMS vs SHAM | Andrew M. Speer 2013 | 0.39 | -0.61 | 1.38 | N.A. |  | Bahadir Bakim 2012 | -1.22 | -2.1 | -0.35 | N.A. |
|  | Paul B. Fitzgerald 2012 | -0.66 | -1.26 | -0.05 | N.A. |  | Shaw-Ji Chen 2018 | -1.86 | -2.91 | -0.82 | N.A. |
|  | Huirong Zheng 2015 | -2.12 | -3 | -1.24 | N.A. |  | Nashaat N. Boutros 2002 | 0.01 | -0.83 | 0.85 | N.A. |
|  | Chris Baeken 2013 | -2.11 | -3.02 | -1.2 | N.A. |  | Urs P. Mosimann 2004 | 0.42 | -0.42 | 1.25 | N.A. |
|  | Hongjun Peng 2012 | -0.30 | -1.16 | 0.57 | N.A. |  | Pooled RR | -0.60 | -1.16 | -0.04 | 65.80% |
|  | Paul E Holtzheime 2004 | -0.23 | -1.24 | 0.79 | N.A. | HFL sub-rTMS vs SHAM | C. Miniussi 2005 | 0.87 | -0.05 | 1.79 | N.A. |
|  | Bahadir Bakim 2012 | -0.99 | -1.86 | -0.12 | N.A. |  | Frank Padberg 1999 | -0.42 | -1.57 | 0.72 | N.A. |
|  | Pooled RR | -0.87 | -1.53 | -0.21 | 75.80% |  | Pooled RR | 0.27 | -0.99 | 1.53 | 66.10% |
| LFR-HFL sup-rTMSvs HFL sup-rTMS | Paul B. Fitzgerald 2018 | -0.32 | -0.94 | 0.30 | N.A. | LFR-HFL sup-rTMS vs SHAM | Minna Valkonen-Korhonen 2018 | -0.01 | -0.66 | 0.63 | N.A. |
|  | Paul B. Fitzgerald 2016 | 0.51 | -0.08 | 1.09 | N.A. |  | Mauro Garcia-Toro 2007 | -0.44 | -1.33 | 0.45 | N.A. |
|  | Pooled RR | 0.10 | -0.71 | 0.91 | 72.30% |  | Paul B. Fitzgerald 2012 | -0.07 | -0.68 | 0.53 | N.A. |
|  | | | | | |  | Paul B. Fitzgerald 2016 | -0.04 | -0.62 | 0.54 | N.A. |
|  |  |  |  |  |  |  | Pooled RR | -0.10 | -0.42 | 0.23 | 0.00% |

**Appendix 5: Assessment of transitivity**

**
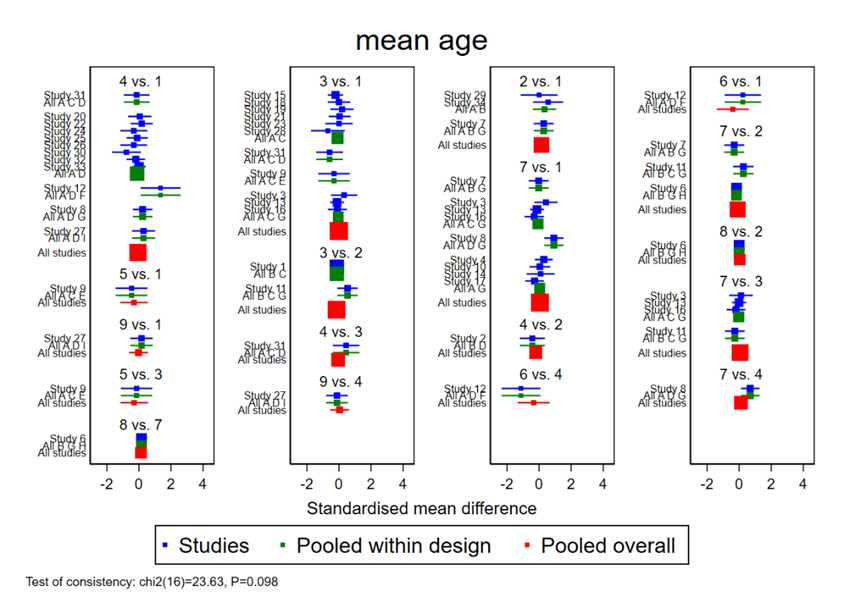
**

**Figure 3：**Although there were a few comparisons which had relatively low or high age means most of the comparisons had similar mean age.

**Appendix 6. Results from network meta-analyses**

**
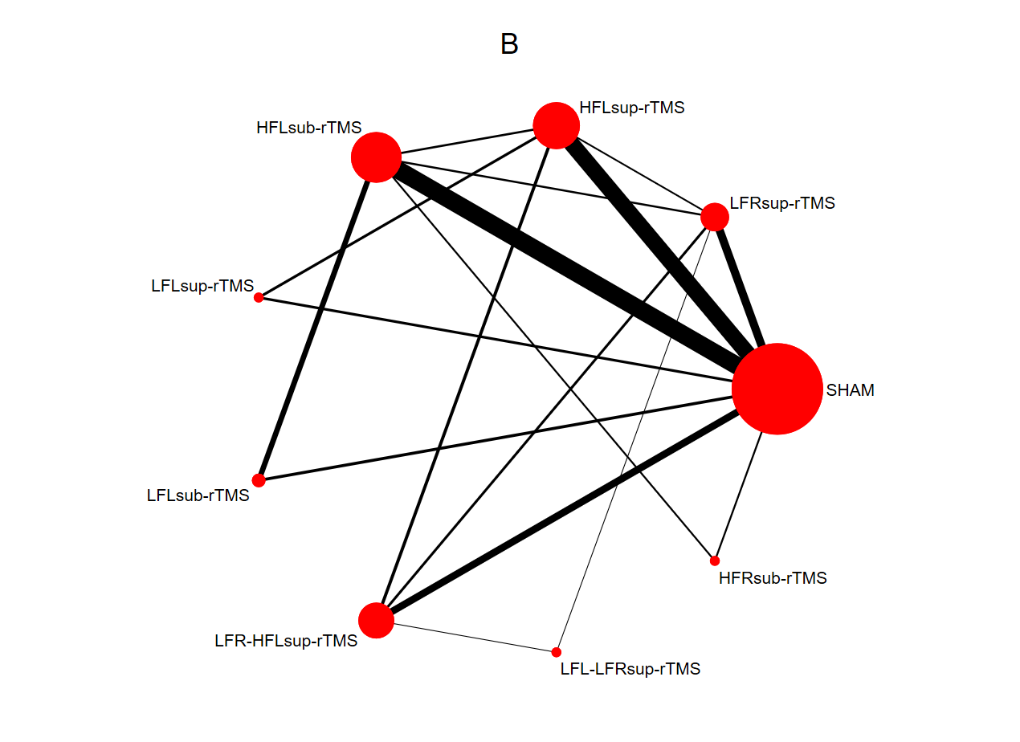

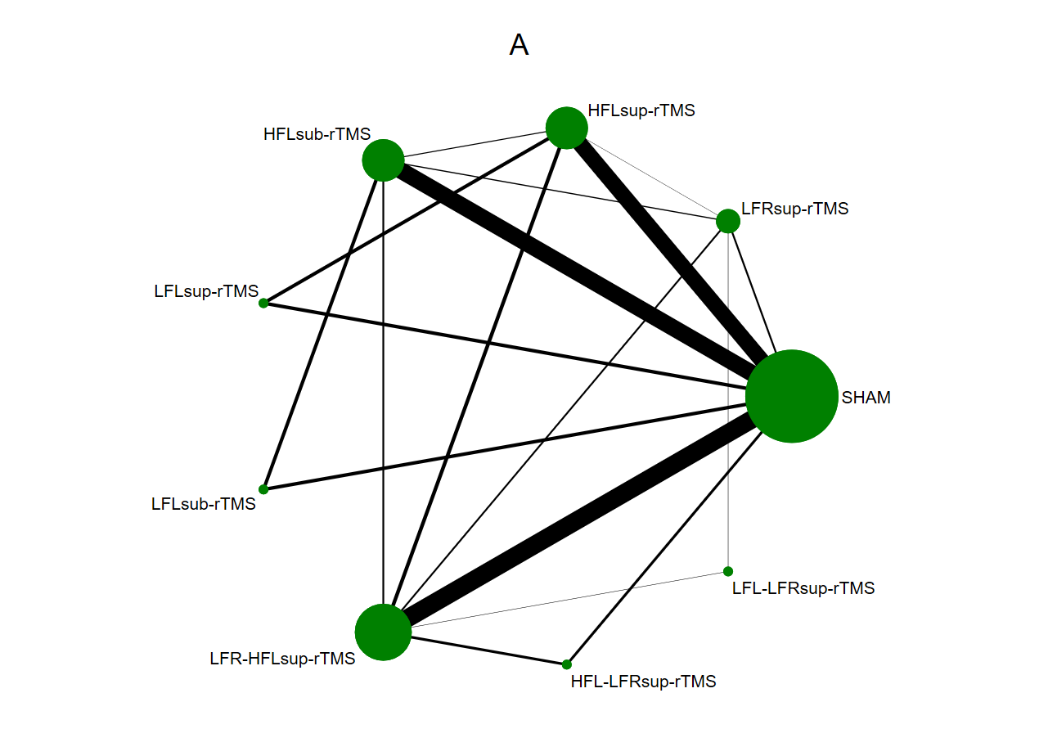
**

**Figure 4:** Network plot of the remission rate (A) and the endpoint depression score (B). Width of the lines represents the number of trials comparing every pair of treatments. Size of every node represents the number of randomly assigned participants.

**Table 8:** Comparisons of the remission rate and endpoint-scores. Pooled RR (95% CI) for remission rates and standardize mean difference (SMD) of endpoint-scores with significant results in bold. RR higher than 1 for remission rate and SMD more than 0 for endpoint-scores favor the first in alphabetical order.

| Remission rate | Endpoint-scores | | | | | | | | | |
| --- | --- | --- | --- | --- | --- | --- | --- | --- | --- | --- |
|  | LFR sup-rTMS | -0.39 (-1.40,0.62) | -0.48 (-1.48,0.53) | -0.76 (-2.67,1.15) | -0.81 (-2.39,0.78) | 0.04 (-0.91,0.99) | —— | -0.03 (-1.57,1.51) | 0.34 (-1.46,2.14) | 0.27 (-0.64,1.18) |
|  |  |  |  |  |  |  |  |  |  |  |
|  | 1.50 (0.57,3.99) | HFL sup-rTMS | -0.08 (-0.93,0.76) | -0.37 (-2.09,1.34) | -0.41 (-1.90,1.07) | 0.43 (-0.43,1.29) | —— | 0.36 (-1.31,2.03) | 0.73 (-0.96,2.43) | **0.66 (0.02,1.30)** |
|  |  |  |  |  |  |  |  |  |  |  |
|  | 1.89 (0.65,5.53) | 1.26 (0.43,3.67) | HFL sub-rTMS | -0.29 (-2.10,1.52) | -0.33 (-1.63,0.96) | 0.51 (-0.42,1.44) | —— | 0.45 (-1.24,2.14) | 0.82 (-0.77,2.40) | **0.75 (0.13,1.36)** |
|  |  |  |  |  |  |  |  |  |  |  |
|  | 2.44 (0.07,89.12) | 1.63 (0.05,54.88) | 1.29 (0.04,47.33) | LFL sup-rTMS | -0.04 (-2.22,2.14) | 0.80 (-1.04,2.64) | —— | 0.73 (-1.60,3.06) | 1.11 (-1.22,3.44) | 1.03 (-0.68,2.75) |
|  |  |  |  |  |  |  |  |  |  |  |
|  | 2.74 (0.08,98.75) | 1.83 (0.05,64.99) | 1.45 (0.04,47.70) | 1.12 (0.01,157.59) | LFL sub-rTMS | 0.84 (-0.69,2.38) | —— | 0.78 (-1.31,2.86) | 1.15 (-0.87,3.17) | 1.08 (-0.28,2.44) |
|  |  |  |  |  |  |  |  |  |  |  |
|  | 1.12 (0.46,2.74) | 0.75 (0.29,1.92) | 0.59 (0.21,1.67) | 0.46 (0.01,16.28) | 0.41 (0.01,14.31) | LFR-HFL sup-rTMS | —— | -0.07 (-1.60,1.47) | 0.31 (-1.44,2.05) | 0.23 (-0.51,0.98) |
|  |  |  |  |  |  |  |  |  |  |  |
|  | 0.43 (0.03,5.73) | 0.29 (0.02,3.79) | 0.23 (0.02,3.07) | 0.18 (0.00,12.80) | 0.16 (0.00,11.22) | 0.38 (0.03,4.56) | HFL-LFR sup-rTMS | —— | —— | —— |
|  |  |  |  |  |  |  |  |  |  |  |
|  | 1.21 (0.33,4.41) | 0.81 (0.18,3.54) | 0.64 (0.14,2.99) | 0.50 (0.01,21.18) | 0.44 (0.01,18.63) | 1.08 (0.30,3.92) | 2.81 (0.18,44.94) | LFL-LFR sup-rTMS | 0.37 (-1.88,2.62) | 0.30 (-1.31,1.91) |
|  |  |  |  |  |  |  |  |  |  |  |
|  | —— | —— | —— | —— | —— | —— | —— | —— | HFR sub-rTMS | -0.07 (-1.66,1.51) |
|  |  |  |  |  |  |  |  |  |  |  |
|  | **3.98 (1.48,10.69)** | **2.65 (1.07,6.53)** | 2.10 (0.84,5.27) | 1.63 (0.05,54.88) | 1.45 (0.04,47.70) | **3.54 (1.60,7.85)** | 9.24 (0.78,109.72) | 3.28 (0.78,13.86) | —— | SHAM |


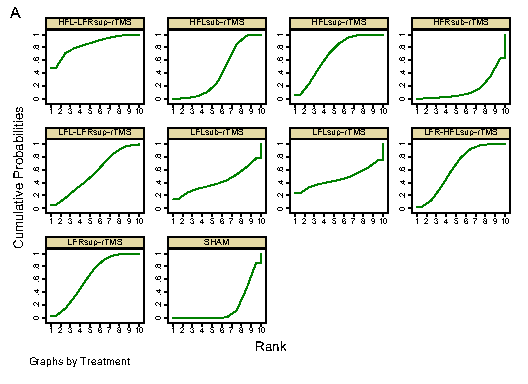

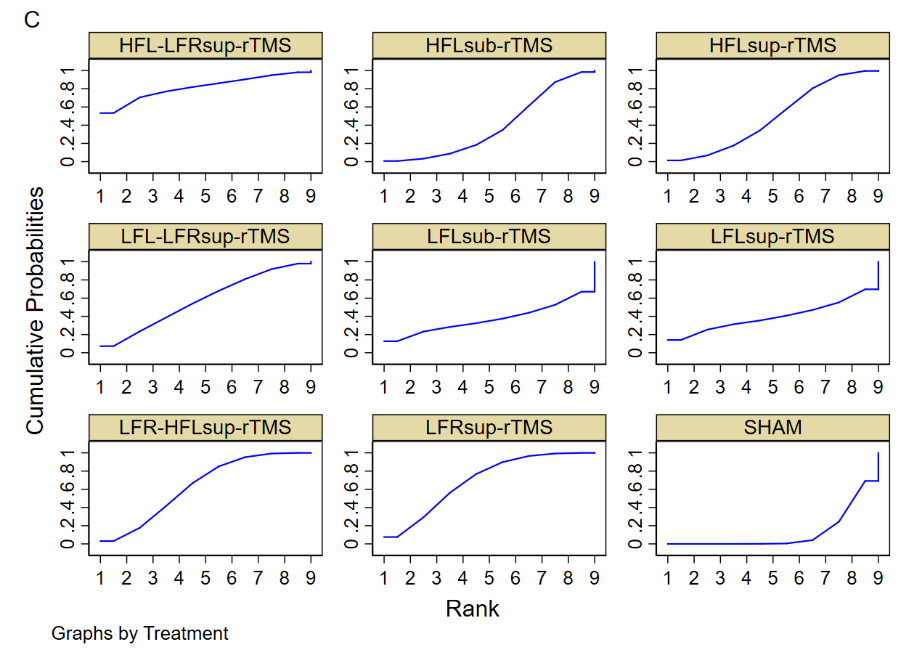

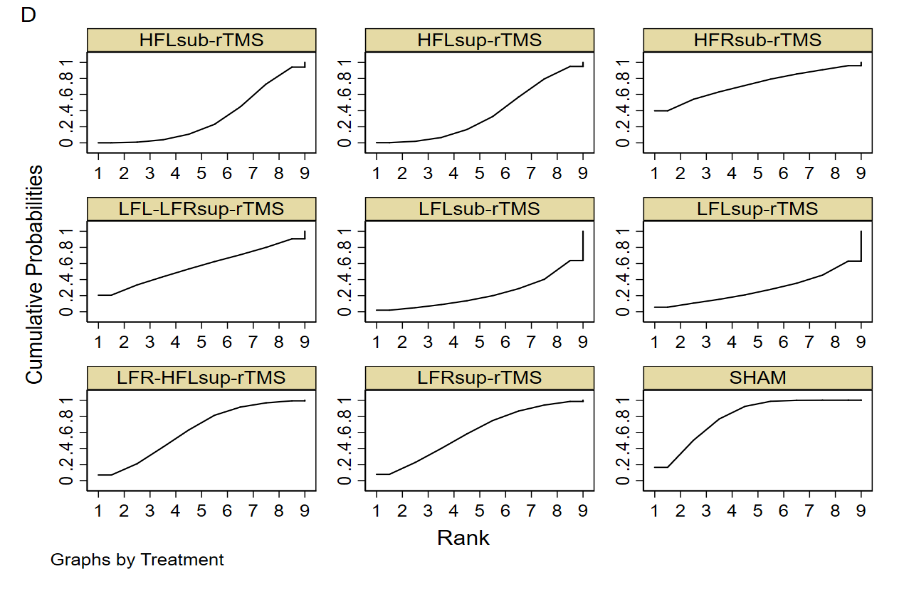

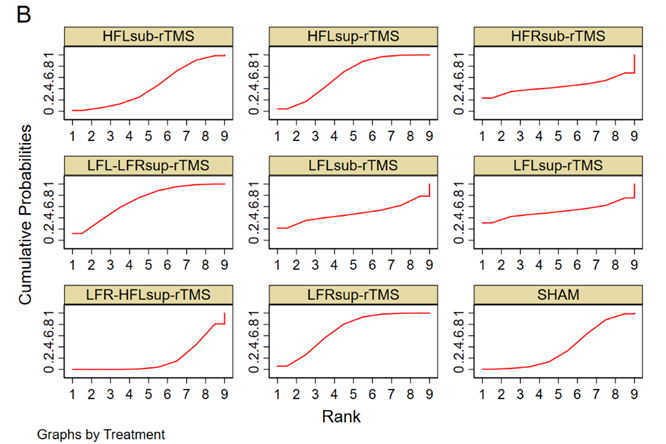


**Figure 5:** Plots of surface under the cumulative ranking curve (SUCRA) results; (A) SUCRA for response rates, (B) SUCRA for Discontinues rates, (C) SUCRA for remission rates, (D) SUCRA for endpoint-scores

**Table 9:** SUCRA values for each treatment measures

| Efficacy (response rates) | | Acceptability (discontinues rates) | | Remissions rates | | Endpoint scores | |
| --- | --- | --- | --- | --- | --- | --- | --- |
| HFL-LFR sup-rTMS | 0.8440 | LFL-LFR sup-rTMS | 0.7060 | HFL-LFR sup-rTMS | 0.8180 | HFL-LFR sup-rTMS | 0.8160 |
| HFL sup-rTMS | 0.7010 | LFR sup-rTMS | 0.7000 | LFR sup-rTMS | 0.6930 | LFR sup-rTMS | 0.6940 |
| LFR-HFL sup-rTMS | 0.6390 | HFL sup-rTMS | 0.6490 | LFR-HFL sup-rTMS | 0.6390 | LFR-HFL sup-rTMS | 0.6360 |
| LFR sup-rTMS | 0.6390 | LFL sup-rTMS | 0.5180 | LFL-LFR sup-rTMS | 0.5830 | LFL-LFR sup-rTMS | 0.5780 |
| LFL-LFR sup-rTMS | 0.5540 | LFL sub-rTMS | 0.4810 | HFL sup-rTMS | 0.4880 | HFL sup-rTMS | 0.4910 |
| LFL sup-rTMS | 0.4690 | HFR sub-rTMS | 0.4420 | LFL sup-rTMS | 0.3950 | LFL sup-rTMS | 0.3990 |
| LFL sub-rTMS | 0.4280 | HFL sub-rTMS | 0.4410 | HFL sub-rTMS | 0.3910 | HFL sub-rTMS | 0.3910 |
| HFL sub-rTMS | 0.4170 | SHAM | 0.3810 | LFL sub-rTMS | 0.3710 | LFL sub-rTMS | 0.3730 |
| SHAM | 0.1570 | LFR-HFL sup-rTMS | 0.1810 | SHAM | 0.1230 | SHAM | 0.1230 |
| HFR sub-rTMS | 0.1520 |  | |  | |  | |

**Appendix 7: Assessment of consistency**


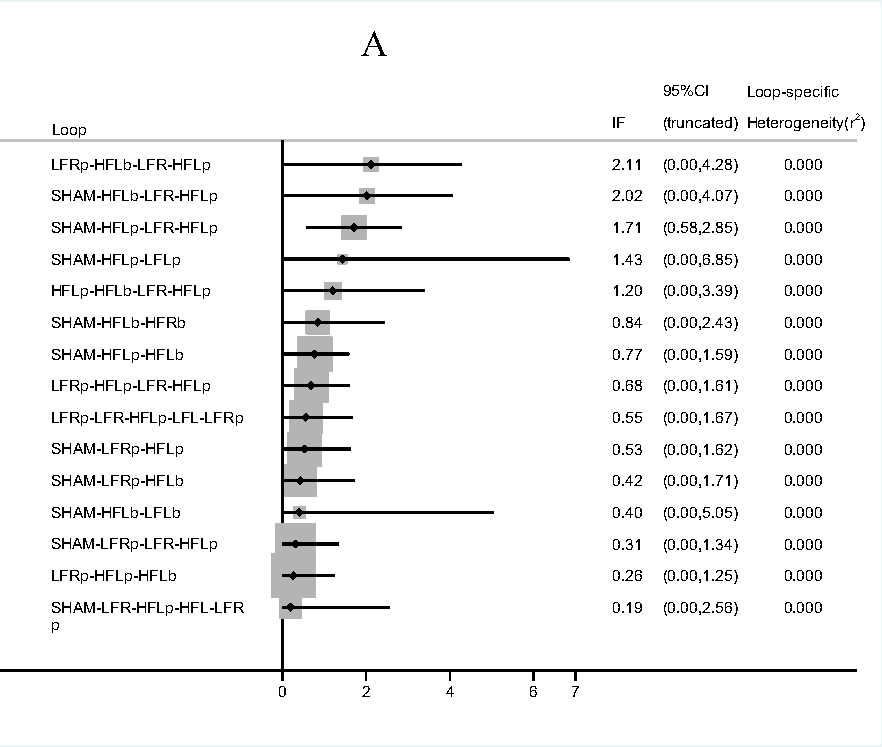

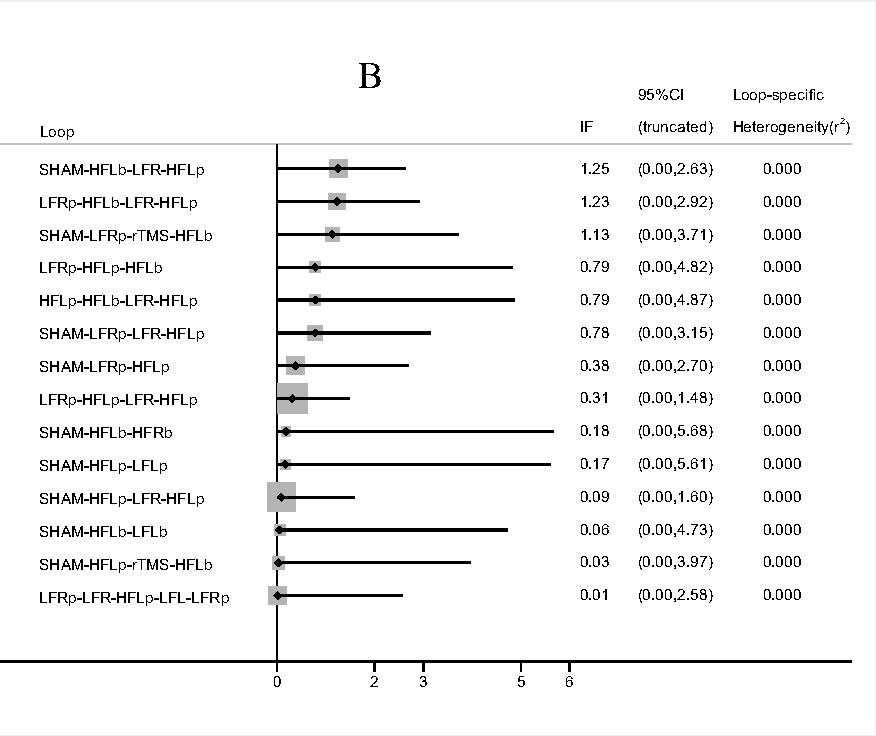

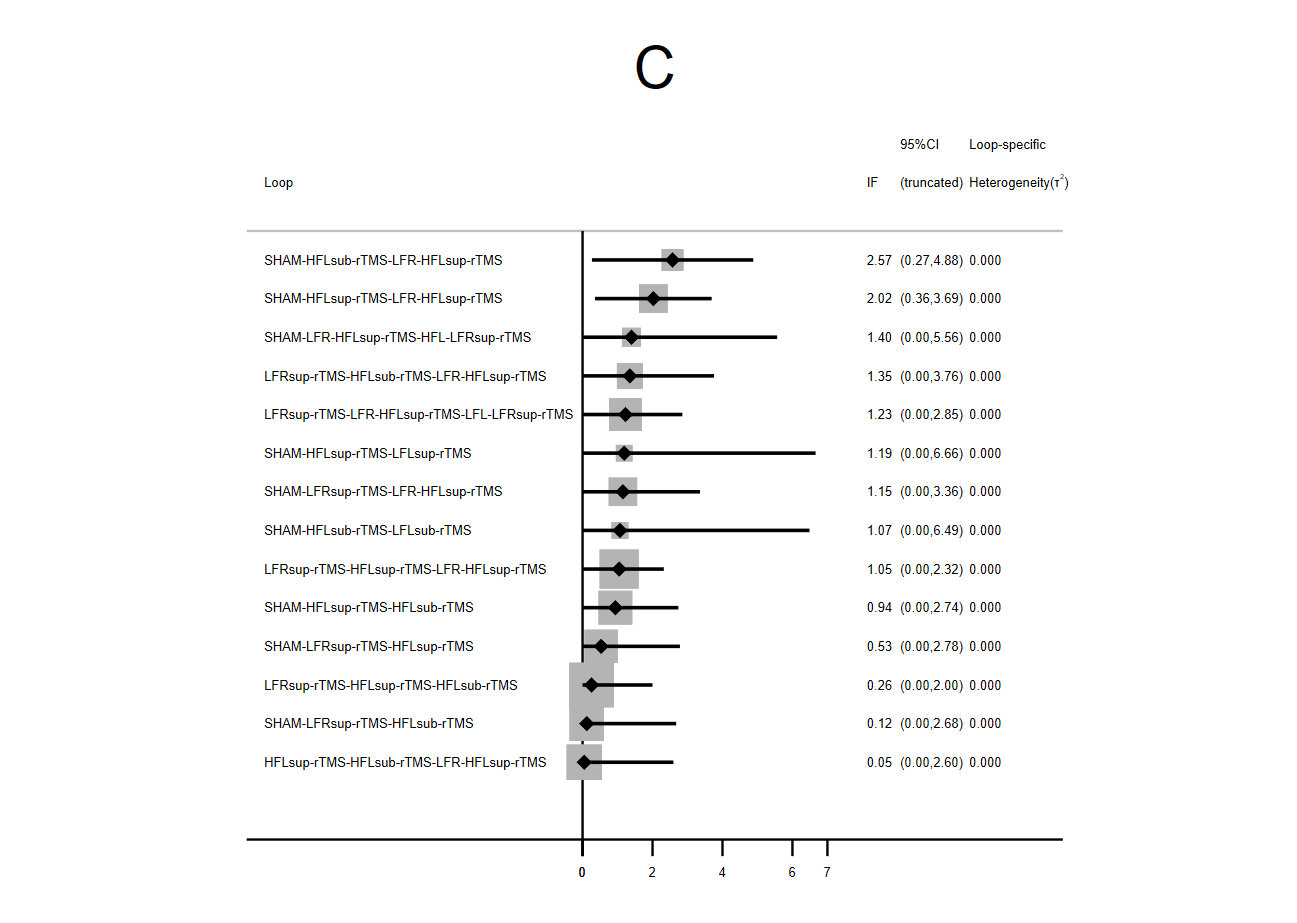

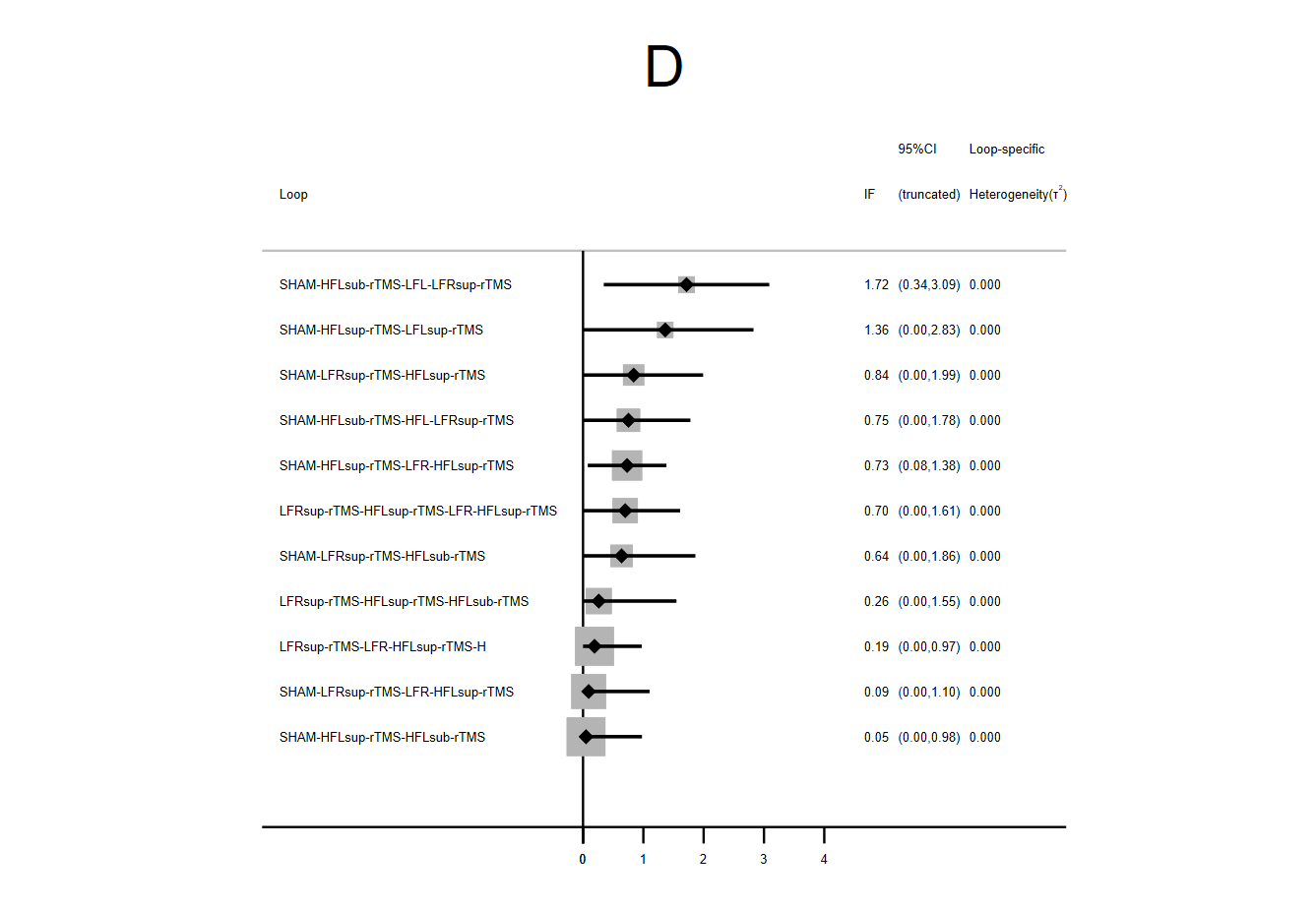


**Figure 6:** (A) Loop inconsistency for efficacy. (B) Loop inconsistency for acceptability. (C) Loop inconsistency for remission rates. (D) Loop inconsistency for endpoint scores. 95% CI did not include 0 were indicative of heterogeneity and the closer inconsistency factor (IF) gets to 0, the less heterogeneity there is.

**Table 9:** Test of heterogeneity with P-values in the global Wald test and the node splitting approach. (A = SHAM, B = LFR sup-rTMS, C = HFL sup-rTMS, D = HFL sub-rTMS, E = LFL sup-rTMS, F = LFL sub-rTMS, G = LFR-HFL sup-rTMS, H = HFL-LFR sup-rTMS, I =LFL-LFR sup-rTMS, J=HFR sub-rTMS)

| Efficacy (response rates) | | | Acceptability (discontinues rates) | | | Remission rates | | | Endpoint scores | | |
| --- | --- | --- | --- | --- | --- | --- | --- | --- | --- | --- | --- |
| global Wald test | 0.926 | | global Wald test | 0.985 | | global Wald test | 0.391 | | global Wald test | 0.986 | |
| The node splitting approach | AB | 0.973 | The node splitting approach | AB | 0.599 | The node splitting approach | AB | 0.491 | The node splitting approach | AB | 0.281 |
|  | AC | 0.201 |  | AC | 0.595 |  | AC | 0.852 |  | AC | 0.266 |
|  | AD | 0.917 |  | AD | 0.808 |  | AD | 0.257 |  | AD | 0.484 |
|  | AE | 0.583 |  | AE | 0.858 |  | AE | 0.628 |  | AE | 0.252 |
|  | AF | 0.756 |  | AF | 0.973 |  | AF | 0.708 |  | AF | 0.200 |
|  | AG | 0.327 |  | AG | 0.216 |  | AG | 0.205 |  | AG | 0.693 |
|  | AH | 0.588 |  | AJ | 0.921 |  | AH | 0.338 |  | AJ | 0.729 |
|  | AJ | 0.129 |  | BC | 0.502 |  | BC | 0.603 |  | BC | 0.345 |
|  | BC | 0.829 |  | BD | 0.280 |  | BD | 0.977 |  | BD | 0.652 |
|  | BD | 0.583 |  | BG | 0.780 |  | BG | 0.645 |  | BG | 0.903 |
|  | BG | 0.545 |  | BI | 0.832 |  | BI | 0.680 |  | BI | 0.848 |
|  | BI | 0.961 |  | CD | 0.920 |  | CD | 0.441 |  | CD | 0.906 |
|  | CD | 0.810 |  | CE | 0.858 |  | CE | 0.628 |  | CE | 0.252 |
|  | CE | 0.583 |  | CG | 0.566 |  | CG | 0.064 |  | CG | 0.459 |
|  | CG | 0.107 |  | DF | 0.969 |  | DF | 0.708 |  | DF | 0.186 |
|  | DF | 0.725 |  | DG | **0.038** |  | DG | 0.147 |  | DJ | 0.729 |
|  | DG | 0.109 |  | DJ | 0.921 |  | GH | 0.338 |  | GI | 0.848 |
|  | DJ | 0.129 |  | GI | 0.832 |  | GI | 0.680 |  |  |  |
|  | GH | 0.588 |  |  |  |  |  |  |  |  |  |
|  | GI | 0.962 |  |  |  |  |  |  |  |  |  |

**Appendix 8: Results of subgroup analysis**

**Table 10:** Comparisons of the efficacy and acceptability for the subgroup <4weeks. Pooled RR (95% CI) for the efficacy and acceptability with significant results in bold. RR higher than 1 for efficacy and less than 1 for acceptability favor the first in alphabetical order.

| Acceptability（discontinuation rate） | | | | | | | | | |
| --- | --- | --- | --- | --- | --- | --- | --- | --- | --- |
| Efficacy (response rate) | LFRsup-rTMS | 0.54 (0.07,3.90) | 0.50 (0.08,3.15) | 0.61 (0.02,24.91) | 0.59 (0.01,23.81) | 0.70 (0.12,4.16) | —— | 0.50 (0.01,21.41) | 0.70 (0.13,3.76) |
|  |  |  |  |  |  |  |  |  |  |
|  | 1.63 (0.33,7.89) | HFLsup-rTMS | 0.92 (0.17,4.87) | 1.14 (0.04,33.49) | 1.09 (0.03,40.47) | 1.30 (0.24,7.10) | —— | 0.92 (0.02,36.43) | 1.30 (0.29,5.72) |
|  |  |  |  |  |  |  |  |  |  |
|  | 1.12 (0.33,3.81) | 0.69 (0.15,3.12) | HFLsub-rTMS | 1.24 (0.04,39.55) | 1.19 (0.04,31.95) | 1.42 (0.36,5.57) | —— | 1.00 (0.03,28.95) | 1.41 (0.66,3.01) |
|  |  |  |  |  |  |  |  |  |  |
|  | 2.10 (0.06,77.43) | 1.29 (0.04,42.13) | 1.88 (0.05,65.84) | LFLsup-rTMS | 0.96 (0.01,107.65) | 1.15 (0.03,38.84) | —— | 0.81 (0.01,95.39) | 1.14 (0.04,33.49) |
|  |  |  |  |  |  |  |  |  |  |
|  | 1.74 (0.05,61.06) | 1.07 (0.03,41.79) | 1.56 (0.05,47.24) | 0.83 (0.01,108.64) | LFLsub-rTMS | 1.19 (0.04,38.93) | —— | 0.84 (0.01,90.60) | 1.19 (0.04,31.95) |
|  |  |  |  |  |  |  |  |  |  |
|  | 1.30 (0.41,4.17) | 0.80 (0.17,3.85) | 1.17 (0.32,4.22) | 0.62 (0.02,23.07) | 0.75 (0.02,26.79) | LFR-HFLsup-rTMS | —— | 0.70 (0.02,24.68) | 0.99 (0.32,3.11) |
|  |  |  |  |  |  |  |  |  |  |
|  | 0.62 (0.11,3.64) | 0.38 (0.05,2.92) | 0.56 (0.09,3.32) | 0.30 (0.01,13.62) | 0.36 (0.01,15.73) | 0.48 (0.11,2.12) | HFL-LFRsup-rTMS | —— | —— |
|  |  |  |  |  |  |  |  |  |  |
|  | 3.02 (0.55,16.56) | 1.86 (0.27,12.63) | 2.70 (0.68,10.76) | 1.44 (0.03,60.76) | 1.73 (0.05,66.23) | 2.31 (0.40,13.28) | 4.85 (0.57,41.26) | HFRsub-rTMS | 1.41 (0.05,40.97) |
|  |  |  |  |  |  |  |  |  |  |
|  | 2.72 (0.99,7.49) | 1.67 (0.44,6.36) | **2.43 (1.19,4.95)** | 1.29 (0.04,42.13) | 1.56 (0.05,47.24) | 2.09 (0.70,6.24) | 4.37 (0.84,22.67) | 0.90 (0.23,3.57) | SHAM |

**Table11:** Comparisons of the efficacy and acceptability for the subgroup ≥4weeks. Pooled RR (95% CI) for the efficacy and acceptability with significant results in bold. RR higher than 1 for efficacy and less than 1 for acceptability favor the first in alphabetical order.

| Efficacy (response rate) | Acceptability（discontinuation rate） | | | | | | |
| --- | --- | --- | --- | --- | --- | --- | --- |
|  | LFRsup-rTMS | 0.97 (0.78,1.20) | 0.88 (0.43,1.77) | 1.01 (0.64,1.58) | 0.88 (0.02,43.11) | **0.47 (0.29,0.76)** | 0.59 (0.33,1.07) |
|  |  |  |  |  |  |  |  |
|  | 0.81 (0.36,1.83) | HFLsup-rTMS | 0.91 (0.45,1.85) | 1.04 (0.64,1.70) | 0.91 (0.02,44.70) | **0.48 (0.29,0.80)** | 0.61 (0.34,1.10) |
|  |  |  |  |  |  |  |  |
|  | 1.63 (0.66,4.01) | 2.00 (0.88,4.52) | HFLsub-rTMS | 1.15 (0.52,2.54) | 1.00 (0.02,46.05) | 0.53 (0.26,1.07) | 0.67 (0.34,1.33) |
|  |  |  |  |  |  |  |  |
|  | 1.07 (0.36,3.19) | 1.32 (0.39,4.39) | 0.66 (0.18,2.36) | LFL-LFRsup-rTMS | 0.87 (0.02,43.50) | **0.46 (0.26,0.81)** | 0.59 (0.29,1.18) |
|  |  |  |  |  |  |  |  |
|  | 1.63 (0.03,97.24) | 2.00 (0.03,117.39) | 1.00 (0.02,54.06) | 1.52 (0.02,100.35) | LFLsub-rTMS | 0.53 (0.01,26.05) | 0.67 (0.01,32.86) |
|  |  |  |  |  |  |  |  |
|  | 0.88 (0.38,2.02) | 1.08 (0.51,2.28) | 0.54 (0.22,1.30) | 0.82 (0.28,2.44) | 0.54 (0.01,32.09) | LFR-HFLsup-rTMS | 1.27 (0.70,2.28) |
|  |  |  |  |  |  |  |  |
|  | **2.58 (1.08,6.16)** | **3.17 (1.60,6.31)** | 1.59 (0.73,3.44) | 2.41 (0.73,7.98) | 1.59 (0.03,92.46) | **2.94 (1.54,5.64)** | SHAM |
|  |  |  |  |  |  |  |  |

**Appendix 9: Sensitivity analysis**

**Table12:** Results of sensitivity analysis in pair meta-analyses. The analyses ware performed by excluding one study at a time and estimated the impact of removing each of the studies on the summary results between HFL sub-rTMS and sham controls.

| Study included at a time | RR | 95% CI | | I^2^ |
| --- | --- | --- | --- | --- |
| Daniel M. Blumberger 2012 | 2.53 | 1.11 | 5.75 | 71.80% |
| Tung-Ping Su 2005 | 2.14 | 0.94 | 4.89 | 71.00% |
| Marie-Laure PaillereMartinot 2009 | 2.26 | 0.92 | 5.53 | 72.30% |
| Nashaat N. Boutros 2002 | 2.49 | 1.07 | 5.78 | 72.00% |
| Mauro Garcia-Toro 2001 | 2.1 | 0.93 | 4.73 | 70.50% |
| Urs P. Mosimann 2004 | 2.27 | 1.01 | 5.13 | 72.10% |
| William J. Triggs 2010 | 2.66 | 1.1 | 6.43 | 69.80% |
| Shaw-Ji Chen 2018 | 2.56 | 1.16 | 5.67 | 55.70% |
| Bahadir Bakim 2012 | 2.11 | 0.9 | 4.92 | 70.40% |
| David Rossini 2005 | 2.01 | 0.92 | 4.42 | 67.90% |
| Christos Theleritis 2017 | 1.72 | 0.93 | 3.17 | 46.90% |


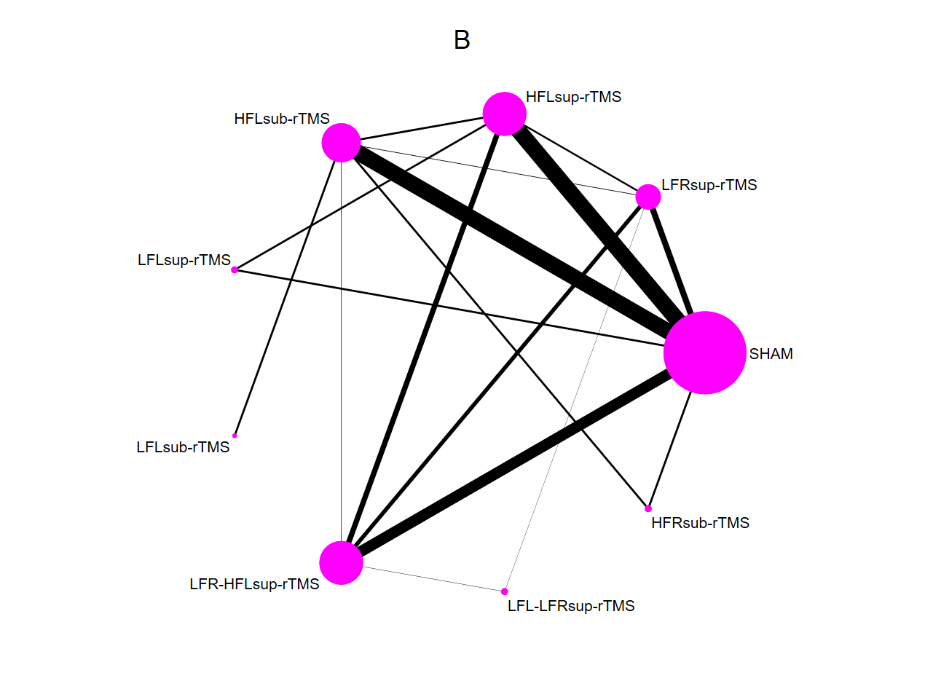

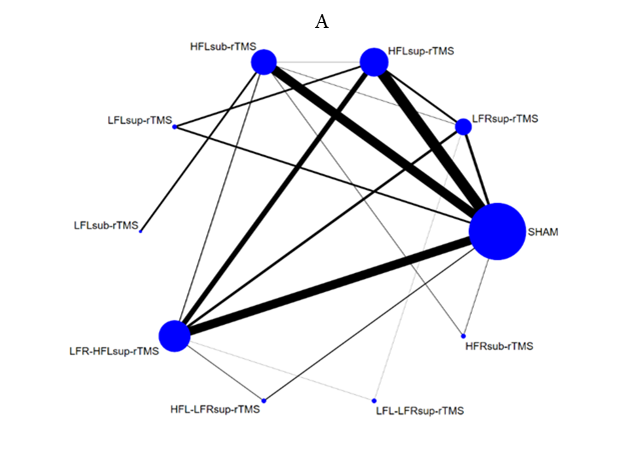


**Figure 7:** Network plot of sensitivity analyses for the efficacy (A) and acceptability (B) in network meta-analyses. Width of the lines represents the number of trials comparing every pair of treatments. Size of every node represents the number of randomly assigned participants.

**Table13:** The comparisons for efficacy and acceptability in sensitivity analyses in network meta-analyses. Pooled RR (95% CI) for acceptability and efficacy, with significant results in bold. RR higher than 1 for efficacy and less than 1 for acceptability favor the first in alphabetical order.

|  | **Acceptability（discontinuation rate）** | | | | | | | | | |
| --- | --- | --- | --- | --- | --- | --- | --- | --- | --- | --- |
| **Efficacy (response rate)** |  |  |  |  |  |  |  |  |  |  |
|  | LFR sup-rTMS | 0.96 (0.78,1.19) | 0.75 (0.41,1.40) | 0.83 (0.03,22.68) | 0.75 (0.02,36.50) | **0.51 (0.32,0.81)** | —— | 1.03 (0.66,1.62) | 0.61 (0.02,18.17) | 0.71 (0.42,1.20) |
|  |  |  |  |  |  |  |  |  |  |  |
|  | 0.91 (0.48,1.72) | HFL sup-rTMS | 0.79 (0.42,1.47) | 0.86 (0.03,23.54) | 0.79 (0.02,38.04) | **0.53 (0.33,0.86)** | —— | 1.08 (0.66,1.75) | 0.64 (0.02,18.93) | 0.74 (0.44,1.25) |
|  |  |  |  |  |  |  |  |  |  |  |
|  | 1.41 (0.72,2.76) | 1.56 (0.83,2.92) | HFL sub-rTMS | 1.10 (0.04,30.83) | 1.00 (0.02,46.05) | 0.68 (0.37,1.24) | —— | 1.37 (0.67,2.82) | 0.82 (0.03,23.39) | 0.94 (0.57,1.56) |
|  |  |  |  |  |  |  |  |  |  |  |
|  | 1.58 (0.05,49.92) | 1.74 (0.06,53.14) | 1.12 (0.04,35.01) | LFL sup-rTMS | 0.91 (0.01,146.80) | 0.62 (0.02,17.17) | —— | 1.25 (0.04,35.23) | 0.74 (0.01,82.69) | 0.86 (0.03,23.54) |
|  |  |  |  |  |  |  |  |  |  |  |
|  | 1.41 (0.03,77.84) | 1.56 (0.03,85.17) | 1.00 (0.02,52.08) | 0.89 (0.00,168.61) | LFL sub-rTMS | 0.68 (0.01,32.65) | —— | 1.37 (0.03,67.51) | 0.82 (0.01,132.76) | 0.94 (0.02,44.84) |
|  |  |  |  |  |  |  |  |  |  |  |
|  | 1.00 (0.55,1.84) | 1.10 (0.59,2.07) | 0.71 (0.37,1.35) | 0.63 (0.02,19.83) | 0.71 (0.01,38.92) | LFR-HFL sup-rTMS | —— | **2.03 (1.17,3.50)** | 1.21 (0.04,35.58) | 1.39 (0.84,2.31) |
|  |  |  |  |  |  |  |  |  |  |  |
|  | 0.52 (0.11,2.38) | 0.57 (0.12,2.63) | 0.37 (0.08,1.68) | 0.33 (0.01,13.36) | 0.37 (0.01,25.34) | 0.52 (0.12,2.16) | HFL-LFR sup-rTMS | —— | —— | —— |
|  |  |  |  |  |  |  |  |  |  |  |
|  | 1.14 (0.45,2.93) | 1.26 (0.44,3.60) | 0.81 (0.28,2.35) | 0.72 (0.02,25.11) | 0.81 (0.01,48.55) | 1.14 (0.45,2.92) | 2.21 (0.40,12.10) | LFL-LFR sup-rTMS | 0.60 (0.02,17.94) | 0.69 (0.36,1.30) |
|  |  |  |  |  |  |  |  |  |  |  |
|  | 3.39 (0.82,14.09) | 3.74 (0.92,15.21) | 2.40 (0.64,9.04) | 2.14 (0.06,83.17) | 2.40 (0.04,155.31) | 3.39 (0.84,13.71) | 6.56 (0.92,46.77) | 2.97 (0.58,15.30) | HFR sub-rTMS | 1.15 (0.04,33.13) |
|  |  |  |  |  |  |  |  |  |  |  |
|  | **2.76 (1.51,5.05)** | **3.04 (1.75,5.29)** | **1.95 (1.19,3.21)** | 1.74 (0.06,53.14) | 1.95 (0.04,104.90) | **2.75 (1.63,4.64)** | **5.33 (1.23,23.13)** | 2.41 (0.88,6.62) | 0.81 (0.22,3.04) | SHAM |
|  |  |  |  |  |  |  |  |  |  |  |


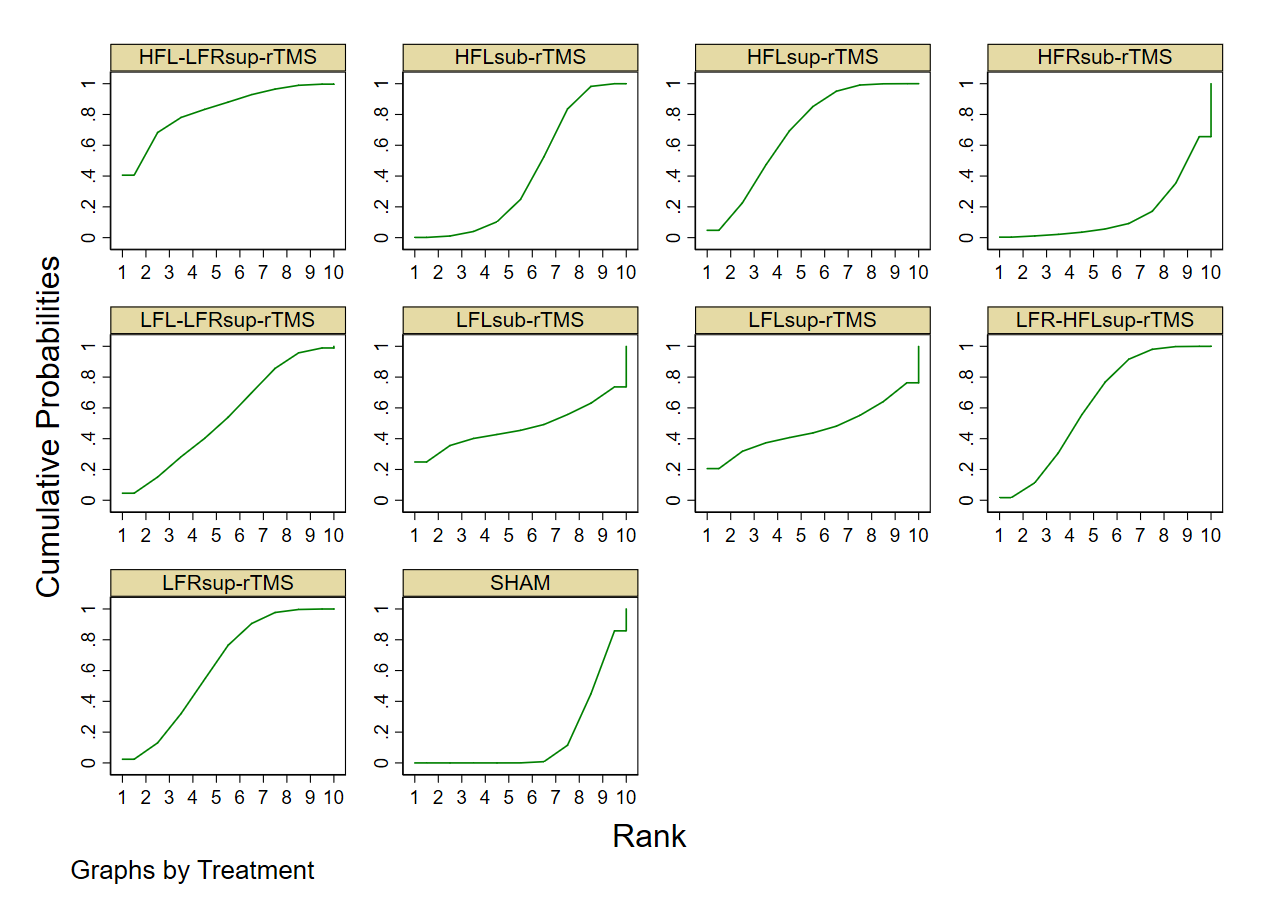

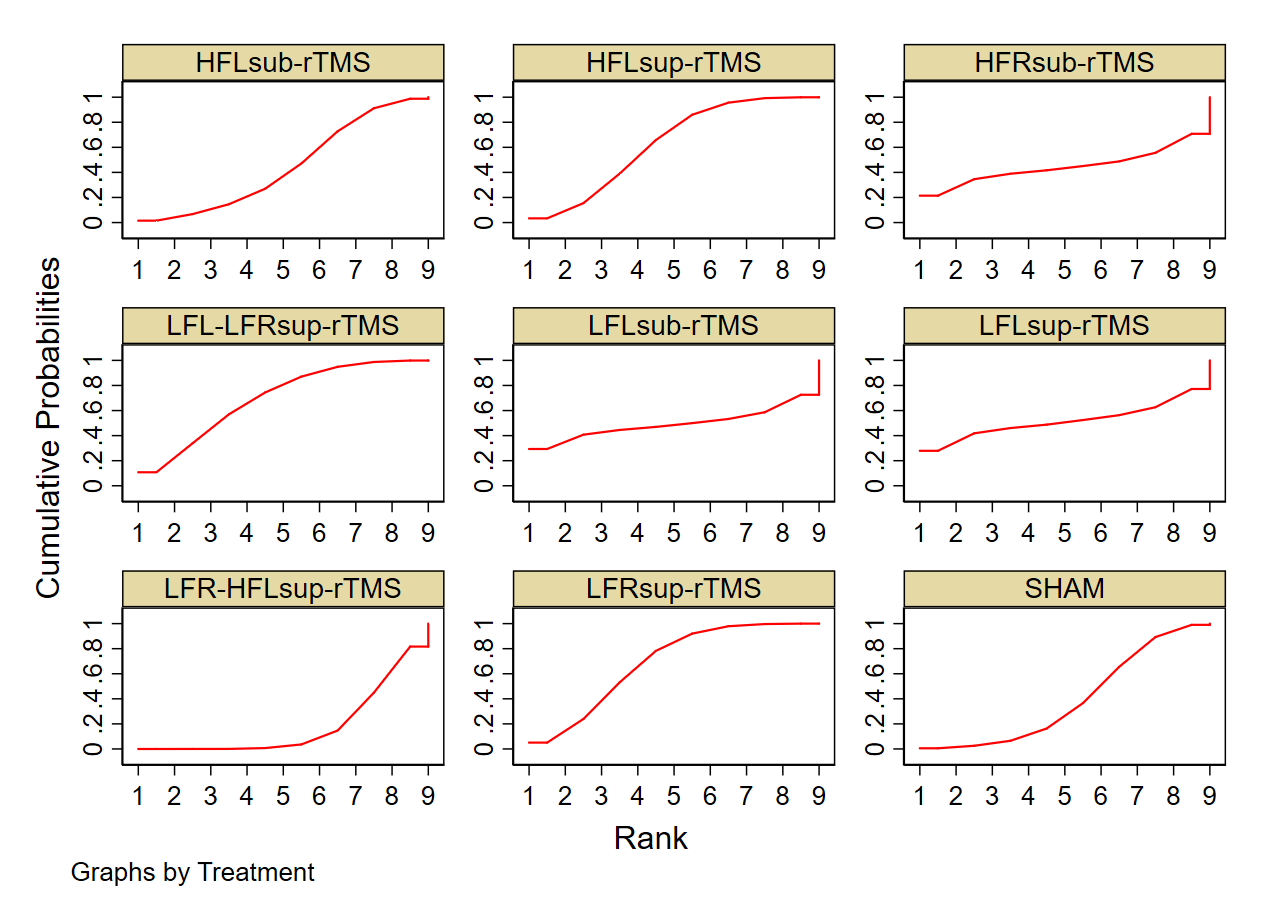

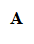

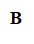


**Figure 8:** Plots of surface under the cumulative ranking curve (SUCRA) results in sensitivity analyses. (A) SUCRA for response rates, (B) SUCRA for Discontinues rates

**Table 14:** SUCRA values for the efficacy and acceptability in sensitivity analyses.

| Efficacy (response rates) | | Acceptability (discontinues rates) | |
| --- | --- | --- | --- |
| HFL-LFR sup-rTMS | 82.90% | LFL-LFR sup-rTMS | 69.60% |
| HFL sup-rTMS | 69.20% | LFR sup-rTMS | 68.80% |
| LFR-HFL sup-rTMS | 62.90% | HFL sup-rTMS | 63.10% |
| LFR sup-rTMS | 62.90% | LFL sup-rTMS | 51.60% |
| LFL-LFR sup-rTMS | 54.70% | LFL sub-rTMS | 49.50% |
| LFL sub-rTMS | 47.80% | HFL sub-rTMS | 45.00% |
| LFL sup-rTMS | 46.40% | HFR sub-rTMS | 44.60% |
| HFL sub-rTMS | 41.60% | SHAM | 39.60% |
| SHAM | 15.90% | LFR-HFL sup-rTMS | 18.20% |
| HFR sub-rTMS | 15.60% |  | |

**Appendix 10: Funnel plot for the efficacy and acceptability of the included treatments**


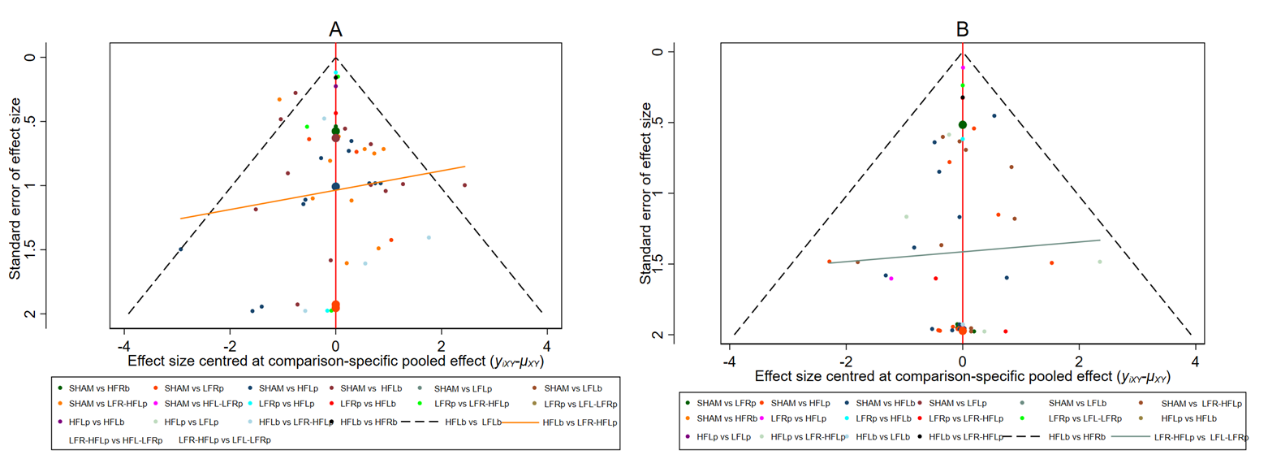


**Figure 9:** (A) Funnel plot for the efficacy of the included treatments. (B) Funnel plot for the acceptability of the included treatments. The effect size is plotted on the horizontal axis, and its standard error is on the vertical axis in the funnel plot. Smaller studies with more significant standard error scatter widely at the bottom of the graph, while the spread narrows among the more extensive studies at the top of the plot.

**Appendix 11: References of included studies**

1. Fitzgerald P B, Hoy K E, Reynolds J, et al. A pragmatic randomized controlled trial exploring the relationship between pulse number and response to repetitive transcranial magnetic stimulation treatment in depression. Brain Stimul. 2020; 13:145-152.
2. Trevizol A P, Goldberger K W, Mulsant B H, et al. Unilateral and bilateral repetitive transcranial magnetic stimulation for treatment-resistant late-life depression. Int J Geriatr Psychiatry. 2019; 34:822-827.
3. Fitzgerald P B, Hoy K E, Elliot D, et al. Exploring alternative rTMS strategies in non-responders to standard high frequency left-sided treatment: A switching study. J Affect Disord. 2018; 232:79-82.
4. Valkonen-Korhonen M, Leinola H, Könönen M, et al. Bifrontal active and sham rTMS in treatment-resistant unipolar major depression. Nord J Psychiatry. 2018; 72:586-592.
5. Theleritis C, Sakkas P, Paparrigopoulos T, et al. Two Versus One High-Frequency Repetitive Transcranial Magnetic Stimulation Session per Day for Treatment-Resistant Depression: A Randomized Sham-Controlled Trial. J ECT. 2017; 33:190-197.
6. Fitzgerald P B, Hoy K E, Elliot D, et al. A negative double-blind controlled trial of sequential bilateral rTMS in the treatment of bipolar depression. J Affect Disord. 2016; 198:158-162.
7. Blumberger D M, Maller J J, Thomson L, et al. Unilateral and bilateral MRI-targeted repetitive transcranial magnetic stimulation for treatment-resistant depression: a randomized controlled study. J Psychiatry Neurosci. 2016; 41: E58-E66.
8. Zheng H, Jia F, Guo G, et al. Abnormal Anterior Cingulate N-Acetylaspartate and Executive Functioning in Treatment-Resistant Depression After rTMS Therapy. Int J Neuropsychopharmacol. 2015; 18: v59.
9. Speer A M, Wassermann E M, Benson B E, et al. Antidepressant efficacy of high and low frequency rTMS at 110% of motor threshold versus sham stimulation over left prefrontal cortex. Brain Stimul. 2014; 7:36-41.
10. Krstić J, Buzadžić, I, Milanović S D, et al. Low-frequency repetitive transcranial magnetic stimulation in the right prefrontal cortex combined with partial sleep deprivation in treatment-resistant depression: a randomized sham-controlled trial. J ECT. 2014; 30:325-331.
11. Chen S J, Chang C H, Tsai H C, et al. Superior antidepressant effect occurring 1 month after rTMS: add-on rTMS for subjects with medication-resistant depression. Neuropsychiatr Dis Treat. 2013; 9:397-401.
12. Baeken C, Vanderhasselt M A, Remue J, et al. Intensive HF-rTMS treatment in refractory medication-resistant unipolar depressed patients. J Affect Disord. 2013; 151:625-631.
13. Blumberger D M, Mulsant B H, Fitzgerald P B, et al. A randomized double-blind sham-controlled comparison of unilateral and bilateral repetitive transcranial magnetic stimulation for treatment-resistant major depression. World J Biol Psychiatry. 2012; 13:423-435.
14. Peng H, Zheng H, Li L, et al. High-frequency rTMS treatment increases white matter FA in the left middle frontal gyrus in young patients with treatment-resistant depression. J Affect Disord. 2012; 136:249-257.
15. Fitzgerald P B, Hoy K E, Herring S E, et al. A double blind randomized trial of unilateral left and bilateral prefrontal cortex transcranial magnetic stimulation in treatment resistant major depression. J Affect Disord. 2012; 139:193-198.
16. Fitzgerald P B, Hoy K, Gunewardene R, et al. A randomized trial of unilateral and bilateral prefrontal cortex transcranial magnetic stimulation in treatment-resistant major depression. Psychol Med. 2011; 41:1187-1196.
17. McDonald W M, Durkalski V, Ball E R, et al. Improving the antidepressant efficacy of transcranial magnetic stimulation: maximizing the number of stimulations and treatment location in treatment-resistant depression. Depress Anxiety.2011; 28:973-980.
18. Paillère M M, Galinowski A, Ringuenet D, et al. Influence of prefrontal target region on the efficacy of repetitive transcranial magnetic stimulation in patients with medication-resistant depression: a [(18)F]-fluorodeoxyglucose PET and MRI study. Int J Neuropsychopharmacol. 2010; 13:45-59.
19. Pallanti S, Bernardi S, Di Rollo A, et al. Unilateral low frequency versus sequential bilateral repetitive transcranial magnetic stimulation: is simpler better for treatment of resistant depression? Neuroscience. 2010; 167:323-328.
20. Zheng H, Zhang L, Li L, et al. High-frequency rTMS treatment increases left prefrontal myo-inositol in young patients with treatment-resistant depression. Prog Neuropsychopharmacol Biol Psychiatry.2010; 34:1189-1195.
21. Fitzgerald P B, Hoy K, Daskalakis Z J, et al. A randomized trial of the anti-depressant effects of low- and high-frequency transcranial magnetic stimulation in treatment-resistant depression. Depress Anxiety.2009; 26:229-234.
22. Triggs W J, Ricciuti N, Ward H E, et al. Right and left dorsolateral pre-frontal rTMS treatment of refractory depression: a randomized, sham-controlled trial. Psychiatry Res. 2010; 178:467-474.
23. Avery D H, Holtzheimer P R, Fawaz W, et al. A controlled study of repetitive transcranial magnetic stimulation in medication-resistant major depression. Biol Psychiatry. 2006; 59:187-194.
24. Garcia-Toro M, Salva J, Daumal J, et al. High (20-Hz) and low (1-Hz) frequency transcranial magnetic stimulation as adjuvant treatment in medication-resistant depression. Psychiatry Res.2006; 146:53-57.
25. Fitzgerald P B, Huntsman S, Gunewardene R, et al. A randomized trial of low-frequency right-prefrontal-cortex transcranial magnetic stimulation as augmentation in treatment-resistant major depression. Int J Neuropsychopharmacol. 2006; 9:655-666.
26. McDonald W M, Easley K, Byrd E H, et al. Combination rapid transcranial magnetic stimulation in treatment refractory depression. Neuropsychiatr Dis Treat. 2006; 2:85-94.
27. Miniussi C, Bonato C, Bignotti S, et al. Repetitive transcranial magnetic stimulation (rTMS) at high and low frequency: an efficacious therapy for major drug-resistant depression? Clin Neurophysiol. 2005; 116:1062-1071.
28. Su T P, Huang C C, Wei I H. Add-on rTMS for medication-resistant depression: a randomized, double-blind, sham-controlled trial in Chinese patients. J Clin Psychiatry. 2005; 66:930-937.
29. Rossini D, Lucca A, Zanardi R, et al. Transcranial magnetic stimulation in treatment-resistant depressed patients: a double-blind, placebo-controlled trial. Psychiatry Res. 2005; 137:1-10.
30. Isenberg K, Downs D, Pierce K, et al. Low frequency rTMS stimulation of the right frontal cortex is as effective as high frequency rTMS stimulation of the left frontal cortex for antidepressant-free, treatment-resistant depressed patients. Ann Clin Psychiatry. 2005; 17:153-159.
31. Holtzheimer P R, Russo J, Claypoole K H, et al. Shorter duration of depressive episode may predict response to repetitive transcranial magnetic stimulation. Depress Anxiety. 2004; 19:24-30.
32. Kauffmann C D, Cheema M A, Miller B E. Slow right prefrontal transcranial magnetic stimulation as a treatment for medication-resistant depression: a double-blind, placebo-controlled study. Depress Anxiety.2004; 19:59-62.
33. Mosimann U P, Schmitt W, Greenberg B D, et al. Repetitive transcranial magnetic stimulation: a putative add-on treatment for major depression in elderly patients. Psychiatry Res. 2004; 126:123-133.
34. Boutros N N, Gueorguieva R, Hoffman R E, et al. Lack of a therapeutic effect of a 2-week sub-threshold transcranial magnetic stimulation course for treatment-resistant depression. Psychiatry Res. 2002; 113:245-254.
35. Garcia-Toro M, Mayol A, Arnillas H, et al. Modest adjunctive benefit with transcranial magnetic stimulation in medication-resistant depression. J Affect Disord. 2001; 64:271-275.
36. Padberg F, Zwanzger P, Thoma H, et al. Repetitive transcranial magnetic stimulation (rTMS) in pharmacotherapy-refractory major depression: comparative study of fast, slow and sham rTMS. Psychiatry Res.1999; 88:163-171.
37. Bakim B, Uzun U E, Karamustafalioglu O, et al. The Combination of Antidepressant Drug Therapy and High-Frequency Repetitive Transcranial Magnetic Stimulation in Medication-Resistant Depression. Klinik Psikofarmakol Bulteni. 2012; 22: 244-253.
